# Identification of Gonadulin and Insulin-Like Growth Factor From Migratory Locusts and Their Importance in Reproduction in *Locusta migratoria*

**DOI:** 10.3389/fendo.2021.693068

**Published:** 2021-06-04

**Authors:** Jan A. Veenstra, Jimena Leyria, Ian Orchard, Angela B. Lange

**Affiliations:** ^1^ INCIA UMR 5287 CNRS, University of Bordeaux, Pessac, France; ^2^ Department of Biology, University of Toronto Mississauga, Mississauga, ON, Canada

**Keywords:** locust, IRP, IGF, ovary, oocytes, fat body

## Abstract

Many insect species have several genes coding for insulin-related peptides (IRPs), but so far only a single IRP gene has been identified in migratory locusts. Here, we report and characterize two other genes coding for peptides that are related to insulin, namely gonadulin and arthropod insulin-like growth factor (aIGF); peptides postulated to be orthologs of *Drosophila melanogaster* insulin-like peptides 8 and 6 respectively. In *Locusta migratoria* the aIGF transcript is expressed in multiple tissues as was previously reported for IRP in both *L. migratoria* and *Schistocerca gregaria*, but there are significant differences in expression patterns between the two species. The gonadulin transcript, however, seems specific to the ovary, whereas its putative receptor transcript is expressed most abundantly in the ovary, fat body and the central nervous system. Since the central nervous system-fat body-ovary axis is essential for successful reproduction, we studied the influence of gonadulin on vitellogenesis and oocyte growth. A reduction in the gonadulin transcript (*via* RNA interference) led to a significant reduction in vitellogenin mRNA levels in the fat body and a strong oocyte growth inhibition, thus suggesting an important role for gonadulin in reproduction in this species.

## Introduction

In insects, the insulin signaling pathway, through insulin-like peptides (ILPs), works as a systemic nutrient sensor regulating processes involved with metabolism and reproduction in accordance with nutritional state (1, 2). In addition, insulin growth factors (IGFs) have been reported in several insect arthropods (aIGFs), which are expressed mainly in the fat body, a regulatory center of nutritional signals, and which have an extended C-terminus. In Diptera, this extended C-terminus is diminished in size and in *Drosophila melanogaster* has been completely lost (3). While the number and type of ILPs varies between species, the elements of the insulin signaling pathway are conserved across the higher invertebrate phyla.

In the coleopteran red flour beetle, *Tribolium castaneum*, four genes encoding ILPs and one for aIGF have been identified in the genome (4), and in the broad-horned flour beetle *Gnatocerus cornutus*, an additional ILP has been described (5). In the Blattodea German cockroach, *Blattella germanica*, several ILPs have been identified. Six of these are expressed mainly in the brain, two in the ovary (Bgilp2 and gonadulin), and a single aIGF expressed in the fat body (3, 6). In two different hemipteran insects, the blood-sucking bug *Rhodnius prolixus* and the migratory brown planthopper *Nilaparvata lugens*, four ILPs have been characterized (7, 8). In *R. prolixus*, one of the ILPs (RhoprIGF) is highly expressed in the fat body and is classified as an aIGF (9), whereas in *N. lugens*, two of them (NlILP2 and NlILP4) resemble IGFs (8). A model insect widely used to study insulin signaling has been the lepidopteran silk moth *Bombyx mori*; and indeed, the first ILP identified in insects was from this species (10). An impressive 38 different ILP genes and a single aIGF gene have been reported in *B. mori* (3, 11, 12), and these influence food intake, energy metabolism and animal growth [reviewed by Mizoguchi and Okamoto (13)].

Major contributions to our understanding of insect ILPs have been obtained using the fruit fly *D. melanogaster*, where eight ILPs (dilps) have been identified. Dilp 6 is produced by the fat body and perhaps by other peripheral tissues and has been referred to as an aIGF (3, 14). A very well conserved peptide across insect species is dilp 7, which has also been called relaxin, and influences lipid synthesis and regulates egg laying decisions (15, 16). Interestingly, dilp 8 is expressed in imaginal discs and coordinates aspects of metamorphosis (17), and is also expressed in both the testis and the ovary (18, 19). Interestingly, although the sequence of this ILP is poorly conserved across species, putative orthologs of dilp8 were recently identified from a variety of arthropods, including the brown marmorated stink bug, *Halyomorpha halys*, the southeastern field cricket, *Gryllus rubens* and *R. prolixus*, and since their transcripts are highly expressed in the ovaries, have been called gonadulins (3, 20).

In addition to binding to receptor tyrosine kinases (RTKs), ILPs can also activate members of the leucine-rich repeat-containing G protein-coupled receptors (LGRs) subfamily of G protein-coupled receptors (GPCRs). For example, dilps 1, 2, 3, 4, 5 and 6 are all believed to act through an RTK, whereas dilp 8 activates an LGR named LGR3 (21). Also, dilp7 was proposed as a ligand for another LGR receptor, LGR4 (22), and recent experimental evidence from *D. melanogaster* suggests this is indeed the case (23). Obviously, this does not exclude that both dilp 7 and dilp 8 might also activate RTK [see *e.g *(24)]. Interestingly, *B*. *mori* lacks clear orthologs for dilps 7 and 8 as well as for their receptors, LGR4 and LGR3, respectively (3). All species for which a gonadulin has been identified also have an ortholog of *Drosophila* LGR3 (3).

Other model species from which ILPs have been identified include orthopterans, where only a single ILP gene has so far been found in the migratory locusts *Locusta migratoria* and *Schistocerca gregaria*. This has been referred to as an insulin-related peptide (IRP) (25–27). Although the genome from *L. migratoria* has been sequenced (28), it has not revealed any additional ILPs (22, 29). Interestingly, *L. migratoria* IRP is expressed in both the neuroendocrine cells of the brain and in the fat body and this is also the case for the *S. gregaria* IRP (26, 30). This is somewhat different from *D. melanogaster*, where the fat body only expresses the aIGF ortholog dilp 6, which is not expressed by the brain neuroendocrine cells. However, in *B. mori* an abundantly expressed fat body ILP is also produced in brain neuroendocrine cells, while aIGF is expressed in a variety of tissues (3, 11, 14, 31).

Although the identification of gonadulin establishes its existence, its physiological relevance remains poorly understood. In addition, as noted above, most of the detailed work on insect ILPs has been done on holometabolous species, where at least the role of the gonadulins may be different from that in hemimetabolous insects. It thus is of interest to examine this putative hormone in a hemimetabolous species, preferably one in which other ILPs have already been studied. Here, we report that migratory locusts lack orthologs of both dilp 7 and its putative receptor, LGR4, but do possess aIGF and gonadulin orthologs. We therefore identify two new peptides likely involved with locust physiology; indeed, we describe the expression of these two transcripts as well as the remarkable effects of gonadulin transcript suppression by dsRNA on vitellogenesis in *L. migratoria*.

## Materials and Methods

### Bioinformatics

We used Artemis (32), Trinity (33) and the SRA Toolkit (https://www.ncbi.nlm.nih.gov/sra/docs/toolkitsoft/) to analyze the *L. migratoria* and *S. gregaria* genome assemblies (28, 34) as well as the *L. migratoria* scaffolds (https://i5k.nal.usda.gov/data/Arthropoda/locmig-(Locusta_migratoria)/Current Genome Assembly/) and various transcriptome SRAs (short read archives) for these species, using methods that have been described in detail elsewhere (35, 36). The following *L. migratoria* transcriptome SRAs were used to identify putative orthologs of aIGF and gonadulin: SRR1283233, SRR1283235, SRR1283237, SRR1283239, SRR1283241, SRR1283243, SRR1283234, SRR1283236, SRR1283238, SRR1283240, SRR1283242, SRR1283244, SRR5754272, SRR5754275, SRR5762723, SRR5762726, SRR5754273, SRR5754276, SRR5762724, SRR5762727, SRR5754274, SRR5754277, SRR5762725 and SRR5762728. For *Schistocerca* we used: DRR018507, SRR6315672 and SRR6315673.

A total of 336 publicly available transcriptome SRAs for *L. migratoria* were used for a preliminary study as to the expression of the previously identified ILP, aIGF, gonadulin and the putative gonadulin receptor, LGR3, in this species using methodology described in detail elsewhere (3, 20). Results are provided in the **Supplementary Spreadsheet** in which numbers in blue refer to the number of spots in each SRA and the number of reads corresponding to each of the four genes. Numbers in black indicate the number of reads per 1,000,000 in each SRA, while the red numbers indicate the ratio of IGF reads to IRP reads.

### Phylogenetic Tree

A phylogenetic tree was made for insect LGRs using published sequences (3) and those constructed here from *L. migratoria* as described. All sequences and their Genbank accession numbers, where available, are listed in the **Supplementary Spreadsheet**. The *L. migratoria* LGR sequences and their orthologs from *S. gregaria* are presented in the **Supplementary Data**. Transmembrane regions were aligned and those were used for tree construction using Fasttree (37) with the following command:./FastTreeDbl -spr 4 -mlacc 2 -slownni -pseudo.

### Animals

The *Locusta migratoria migratorioides* (Reiche and Fairmaire, 1849) colony was reared under crowded conditions at 30°C and 50% humidity at the University of Toronto Mississauga (ON, Canada). Locusts were kept on a 12 h:12 h light:dark regime, and were fed fresh wheat seedlings and bran, ad libitum every day.

### Quantitative Real-Time PCR (RT-qPCR)

The spatial distribution of*, aIGF, IRP*, *gonadulin* and the putative gonadulin receptor, *LGR3*, was carried out on 3-4-week-old sexually mature female *L. migratoria*. Midgut, brain, suboesophageal ganglion, flight muscle, fat body, ovaries, salivary gland, and ventral nerve cord (these last 2 tissues have some difficult-to-remove associated fat body) were dissected under cold phosphate buffered saline autoclaved (PBS, 6.6 mM Na_2_HPO_4_/KH_2_PO_4_, 150 mM NaCl, pH 7.4). In addition, testes from 3-4-week-old males (mature testis) and from 1-2-day-old males (immature testes), as well as ovaries of 1-2-day-old females (immature ovaries) were included. RNA extraction was performed with Trizol reagent (Invitrogen by Thermo Fisher Scientific, MA, USA) according to the manufacturer’s instructions. All samples showed an A260/280 ratio between 1.9 and 2.0 and RNA integrity was evaluated by electrophoresis in a 1% agarose gel (FroggaBio Inc., Concord, ON, Canada). cDNAs were synthesized from 1 µg of total RNA by reverse transcription reaction using random primers and 50 U of MultiScribe™ MuLV reverse transcriptase (High-Capacity cDNA Reverse Transcription Kit, Applied-Biosystems, from Fisher Scientific, ON, Canada). qPCRs were performed using an advanced master mix with super green low rox reagent (Wisent Bioproducts Inc, QC, Canada), according to the manufacturer’s recommendations, using 4 pmol of sense and antisense primers in a final volume of 10 μl. The qPCR temperature-cycling profile was: initial denaturation 3 min at 95°C, followed by 39 cycles of 30 s at 94°C, 30 s at 58–60°C (depending on the pair of primers used), and 1 min at 72°C, followed by a final extension at 72°C for 10 min. At least three independent experiments were performed (n = 3) with each sample composed of tissues from 2-3 insects. Each reaction contained two technical replicates as well as a no template control and a no reverse transcriptase control. qPCR was carried out using a CFX384 TouchTM Real-Time PCR Detection System (BioRad Laboratories Ltd., Mississauga, ON, Canada). The sequences of primers used for amplification (Sigma-Aldrich, ON, Canada) and the efficiencies are shown in the **Table 1**. For each pair of primers, a dissociation curve with a single peak was seen, indicating that a single cDNA product was amplified. Succinate dehydrogenase, ribosomal protein 49, which were previously validated for transcript expression by qPCR in different tissues of locusts (39), were used as housekeeping genes. The results, *i.e.* Cq of each reaction, were analyzed by the 2^-ΔΔCt^ method (40). Transcript expression was normalized *via* geometric averaging taking into account the levels of the housekeeping genes listed above.

**Table 1:** List of gene-specific primers.

Gene	Forward primers (5´—> 3´)	Reverse primers (5´—> 3´)	Efficiency	Source
**RT-qPCR**				
Gonadulin	CTCTACGTGGCAGTCCTGGT	CAGGCGTTCTTCATCAGCTC	0.90	designed here
aIGF	CAGCTACTGCAAGTCCGACA	AACAGCTCGTCTCCGTCCT	1.01	designed here
IRP	GTCCGACCTGTTCCTCCTGT	CCCGCTCCAGTAGTTGTCTT	0.91	designed here
LGR3	ATCAGCCTCGCCGTCATACC	ATACTGCCAGCCGTGGAAGAA	0.92	Zheng et al. (38),
Succinate dehydrogenase	CCACTGAAACTGATCCAAGAGAG	TCCTGCTCCATTAACTAAGCAAC	1	Yang et al. (39),
Ribosomal protein L32	ACTGGAAGTCTTGATGATGCAG	CTGAGCCCGTTCTACAATAGC	0.98	Yang et al. (39),
**dsRNA**				
dsGonadulin	*taatacgactcactatagggagaGCATCCTGGAGCTGATGAAG	*taatacgactcactatagggagaGGTGATCAGCTGGTGGAACT	not applicable	designed here

*taatacgactcactatagggaga–>T7 RNA polymerase promotor.

### Double-Stranded RNA Design and Synthesis

RNA interference using double-stranded RNA (dsRNA) to down-regulate gonadulin mRNA was performed to evaluate gonadulin’s potential physiological role in reproduction. Gonadulin dsRNA (dsGON) was synthesized using the T7 Ribomax Express RNAi System (Promega, WI, USA), according to the manufacturer protocol, and ovarian tissue as template. Gene specific primers (GSPs) were combined with GSPs containing the T7 RNA polymerase promoter sequence (**Table 1**). As an experimental control, a dsRNA based on the Ampicillin Resistance Gene *(*dsARG) from the pGEM-T Easy Vector system (Promega, WI, USA) was used throughout the study (38).

### Knockdown of Transcript Expression Using Double Stranded RNA

To knockdown the expression of *gonadulin*, females 1 day post-ecdysis into the adult stage were separated into two groups. The females of each group were injected into the hemocoel with 15 μg of dsGON or dsARG (control) diluted in 5 μL of ultrapure water using a Hamilton micro syringe, and a second injection was performed on day 5 post-ecdysis. Ten days after the first injection, the phenotype as well as the size of the ovary and the primary follicles were examined and the relative expression in the ovary of *gonadulin* was determined by RT-qPCR, as described above. In addition, to assess the potential effects of dsGON on the expression of yolk protein precursors, fat body was dissected at the same time and vitellogenin (Vg) mRNA expression was evaluated by RT-qPCR. For RNAi experiments, the results are shown as the mean ± standard error of the mean (SEM; n = 14-16, where each sample represents one insect).

### Ovary and Ovariole Size Examination and Data Analysis

Ovaries and ovarioles were photographed with a Leica DVM6 digital microscope (Leica Microsystems, Wetzlar, Germany). The pictures were then used to measure width and length of ovaries and primary follicles to produce ovary and primary follicle size indexes. The ovary size index was obtained by multiplying the length by the width (mm^2^) of each ovary. For follicles the index was defined as length (in mm) x width (in mm)^2^ x π/4 which yields a volume in mm^3^. The primary follicle index is the average of three randomly selected primary follicles of each insect.

### Statistical Analyses

Graphs were created using GraphPad Prism 9 (GraphPad Software, CA, USA, www.graphpad.com). Multiple group analysis was conducted by one-way ANOVA and Tukey´s test as post-hoc test. Statistically significant difference between two groups were inferred using Student’s T-test. A *P* value < 0.05 was considered statistically significant.

## Results

**Figure 1:**
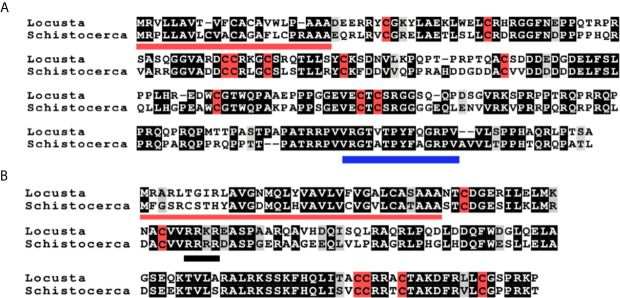
Deduced amino acid sequences of *L. migratoria* and *S. gregaria* aIGFs and gonadulins. **(A)** aIGF sequences, predicted signal peptides are underlined in red, cysteine residues are highlighted in red, other identical amino acids in black and relatively conserved amino acid changes in grey. The C-terminal part of the sequence is somewhat conserved between insect aIGFs and has been underlined in blue. **(B)** Gonadulin sequences, predicted signal peptides are underlined in red, cysteine residues are highlighted in red, other identical amino acids in black and relatively conserved amino acid changes in grey. A possible tetrabasic furin cleavage site is underlined in black.

**Figure 2:**
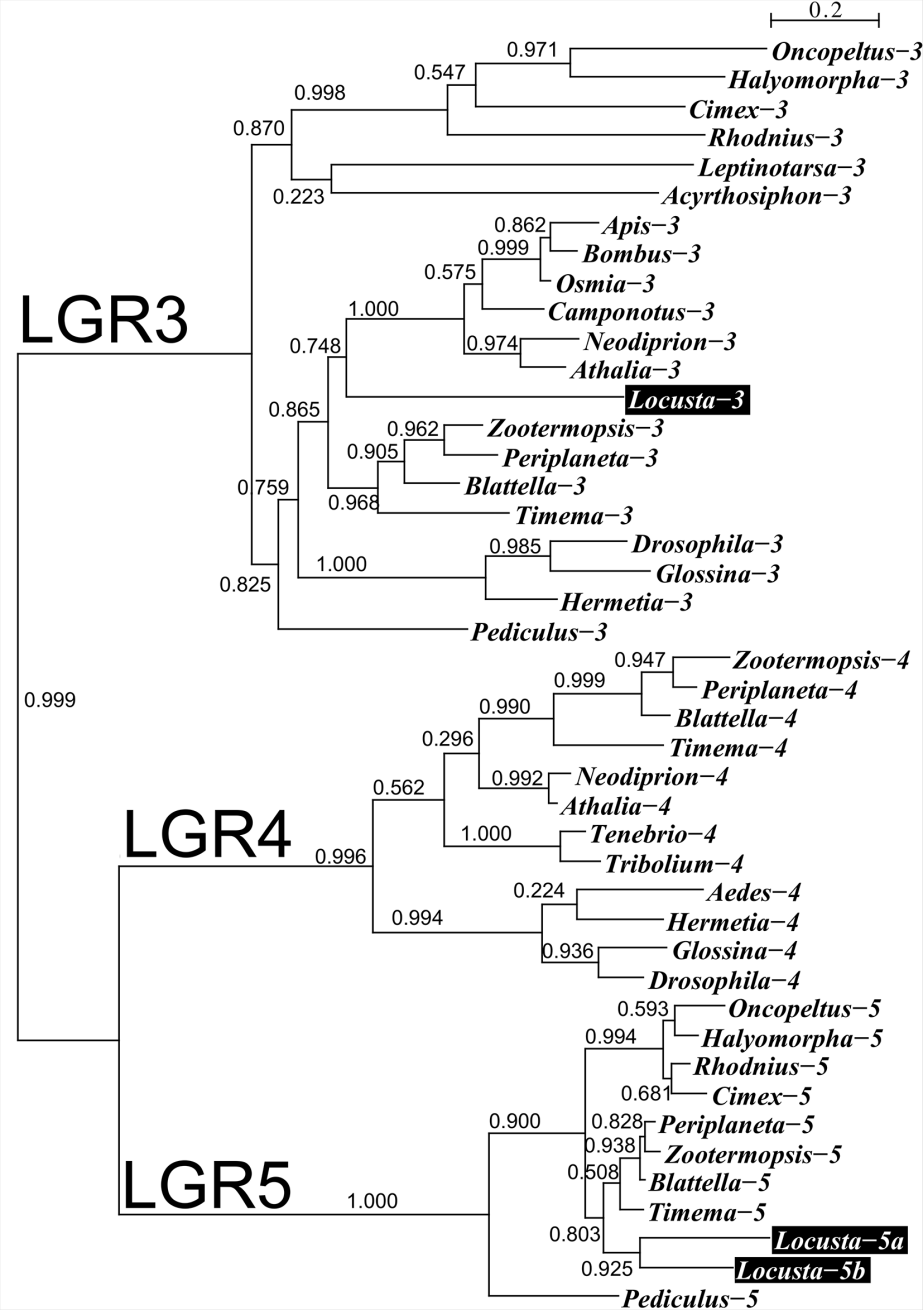
Phylogenetic tree of LGRs 3, 4 and 5 from several hemimetabolous insect species. Note that *L. migratoria* has no LGR4, but one LGR3 and two LGR5 orthologs. The *L. migratoria* LGR3 has previously been suggested to be an LGR4 ortholog (38). Genbank identifiers and sequences are listed in the **Supplementary Spreadsheet**.

We used genome and transcriptome sequences to search for three ILPs in migratory locusts, namely aIGF, relaxin and gonadulin as well as the three insect LGRs that might function as receptors for these peptides. Two novel ILPs were identified from *L. migratoria* and *S. gregaria* transcriptomes; aIGF and gonadulin (**Figures 1A, B**), but both species lack a dilp 7 ortholog. Three different LGRs related to *D. melanogaster* LGR3 and LGR4 were identified in both locust species (**Supplementary Data**). One of these has been previously reported from *L. migratoria* as an LGR4 ortholog (10) however that receptor is not an ortholog of LGR4 but of LGR3 (**Figure 2**); its sequence is also slightly longer than reported by Zheng et al. (38) (**Supplementary Data**). Both *L. migratoria* and *S. gregaria* have two LGR5 orthologs, while other insect species have either one or none (**Figure 2** and **Supplementary Data**). There is less publicly available transcriptome data for *S. gregaria* than there is for *L. migratoria*, but both aIGF and gonadulin could be identified from transcriptome SRAs. Genes coding these peptides as well as LGR3 and the two LGR5s, but not LGR4, are present in the genome assembly of *S. gregaria* (**Supplementary Data**).

The locust aIGFs have ten cysteine (Cys) residues, four more than the typical ILP, and are hence predicted to have five disulfide bridges. These locust sequences differ significantly from those of other insect aIGFs, yet are sufficiently similar to identify them as such. In particular, the poorly conserved sequence at the C-terminus of the molecule is conserved in both species (**Figures 1A, B** and **Supplementary Figure S1**). The structure of IGF is typically considered to be made up of the A-, B-, C-, D- and E-domains, but it is not possible to delineate those in locust aIGF, even though its structure is very similar to IGF of which it is likely an ortholog. The sequence homology with the IRPs is insufficient to identify the limits of the C-domain. Also, there is a lack of a recognizable convertase cleavage site that would indicate the border between of the D- and E-domains, suggesting that the D-domain is very large and the E-domain might be lacking (**Figure 1A**).

**Figure 3:**
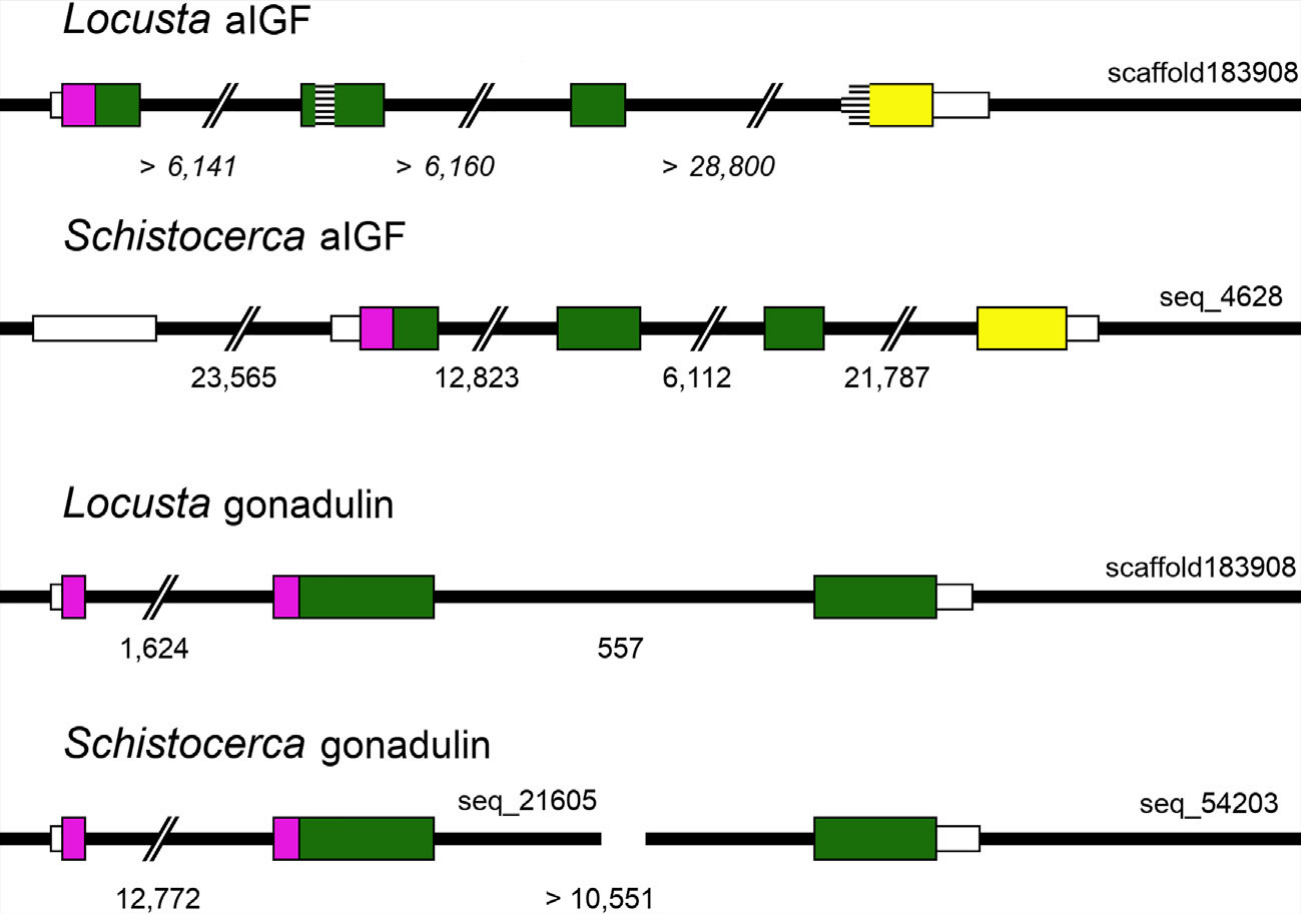
Locust aIGF and gonadulin genes. Thick black lines indicate DNA sequences, colored boxes represent translated exons and smaller white boxes are untranslated sequences. Purple is used to indicate the location of the signal peptide coding sequences, yellow for the conserved C-terminal part of aIGF and green for the remainder of the precursors. Numbers below each drawing indicate the intron sizes, those of the *L. migratoria* aIGF gene are in italic, as the scaffold contains numerous sequence gaps, and the accuracy of the numbers is therefore highly doubtful. Two sequence gaps inside the coding sequence of the *L. migratoria* aIGF gene are indicated by horizontal lines. Scaffold identifiers are also indicated. Note that the *L. migratoria* aIGF and gonadulin genes are located on the same scaffold.

The genomic *L. migratoria* sequence for aIGF has two sequence gaps (**Figure 3**), with the second one covering the four typical Cys residues in the A chain, which readily explains why this peptide was missed in previous analyses of the neuropeptide genes in this species (21, 28), while the *S. gregaria* gene is complete in the current genome assembly. The aIGF genes have four coding exons, like in other species (**Figure 3**). In several insect species the aIGF gene is alternatively spliced leading to two different aIGF isoforms (3), but in locusts no evidence for such alternative splicing was found.

**Figure 4:**
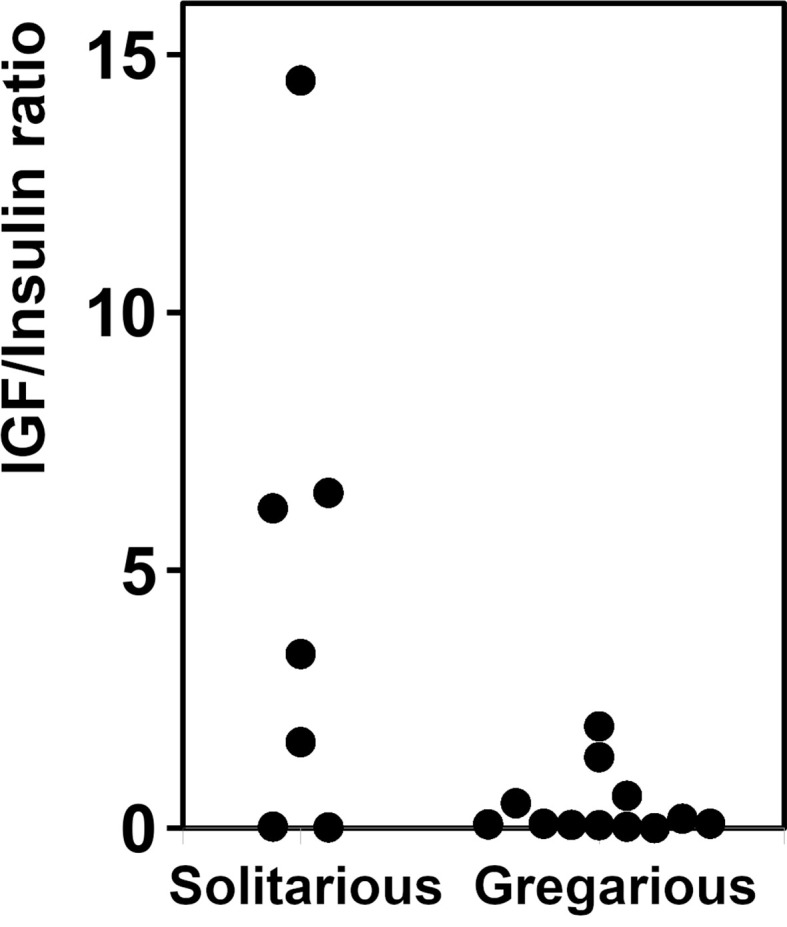
Relative expression of aIGF and IRP in 4^th^ instar *L. migratoria*. Ratio of aIGF reads to IRP reads in transcriptome SRAs from 4^th^ instar *L. migratoria* suggesting that there may be significant differences between solitarious versus gregarious insects.

As there are a large number of publicly available SRAs for *L. migratoria*, we used those to get a preliminary idea of the expression of ILP genes in this species. Analysis of the publicly available SRAs indicates that both *L. migratoria* aIGF and IRP are expressed in multiple tissues (**Supplementary Spreadsheet**), as previously reported for IRP from *S. gregaria* (25) and to a lesser degree also for *L. migratoria* (29). Nevertheless, the data suggested that in *L. migratoria* there are differences in expression between the two peptides and this was confirmed by our profile of expression by RT-qPCR (see below). The ratio of reads corresponding to the expression of aIGF to IRP reads is quite variable. For example, the SRA data suggests that in the ovary the expression of aIGF is more important than that of IRP, while in the flight muscle it is the other way around. In most cases, the number of data points is small, thus precluding any firm conclusions. There is, however, a relatively large number of SRAs for the 4^th^ instars of both solitarious and gregarious locusts. This is the stage where insect density strongly affects whether insects develop gregarious or solitarious phase characteristics. These data reveal an interesting tendency for a higher expression of aIGF in solitarious versus gregarious locusts (**Figure 4**).

**Figure 5:**
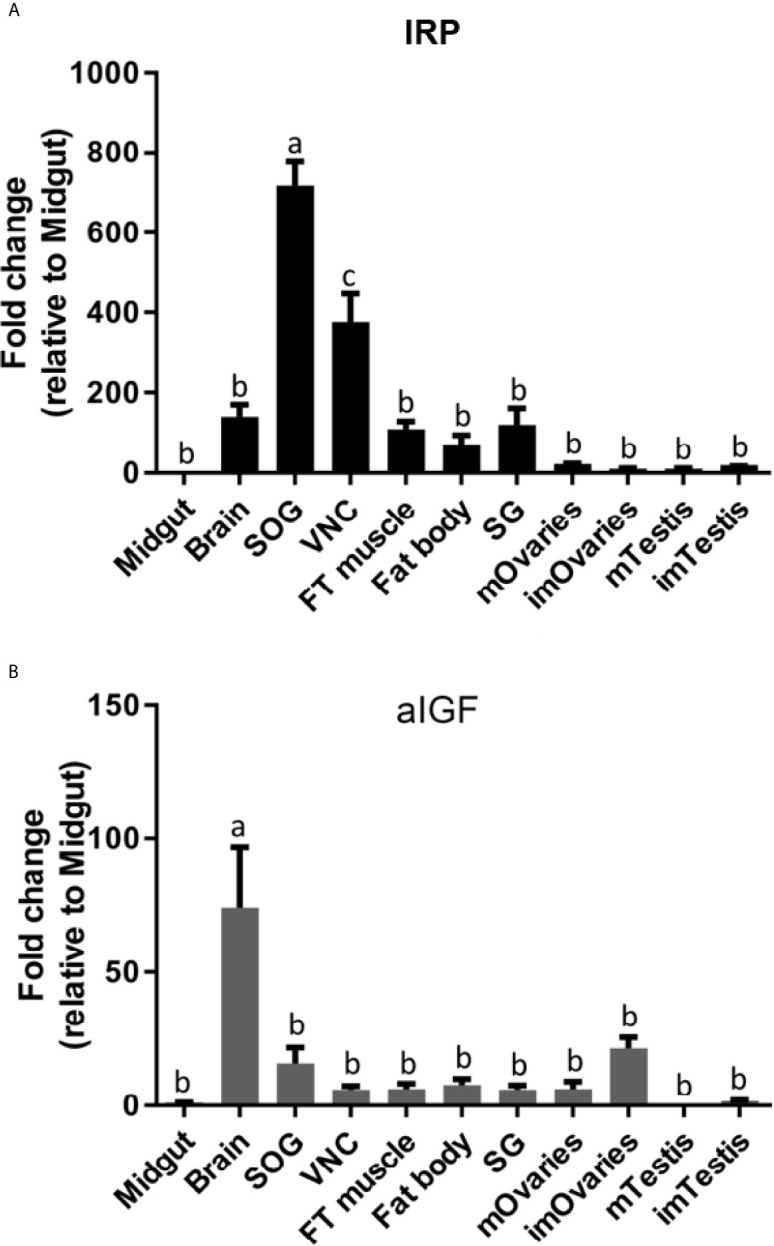
Distribution of IRP **(A)** and aIGF **(B)** transcripts in different tissues from *L. migratoria* females, as well as testes from males. Midgut, brain, suboesophageal ganglion (SOG), flight muscle (FT muscle), salivary glands (SG), ventral nerve cord (VNC), fat body and ovaries (mOvaries) from 3-4-week-old sexually mature female, as well as testes from 3-4-week-old (mature tissue, mTestis), 1-2-days-old males (immature tissue, imTestis), and ovaries of 1-2-days-old females (immature ovaries, imOvaries) were included. The y-axis represents the fold change in expression relative to midgut (value ~ 1). The results are shown as mean ± SEM (n =3, with each n composed of tissues from 2-3 insects). Significance of p < 0.05 is denoted using letters to indicate bars that are significantly different from others.

Given both the structural and physiological similarities of IRP and aIGF we compared the expression of these two transcripts in adult *L. migratoria.* The results confirm their ubiquitous expression, but also some interesting differences. Thus, whereas the brain shows the highest expression of aIGF, it is the suboesophageal ganglion that seems to have the highest IRP expression (**Figures 5A, B**).

**Figure 6:**
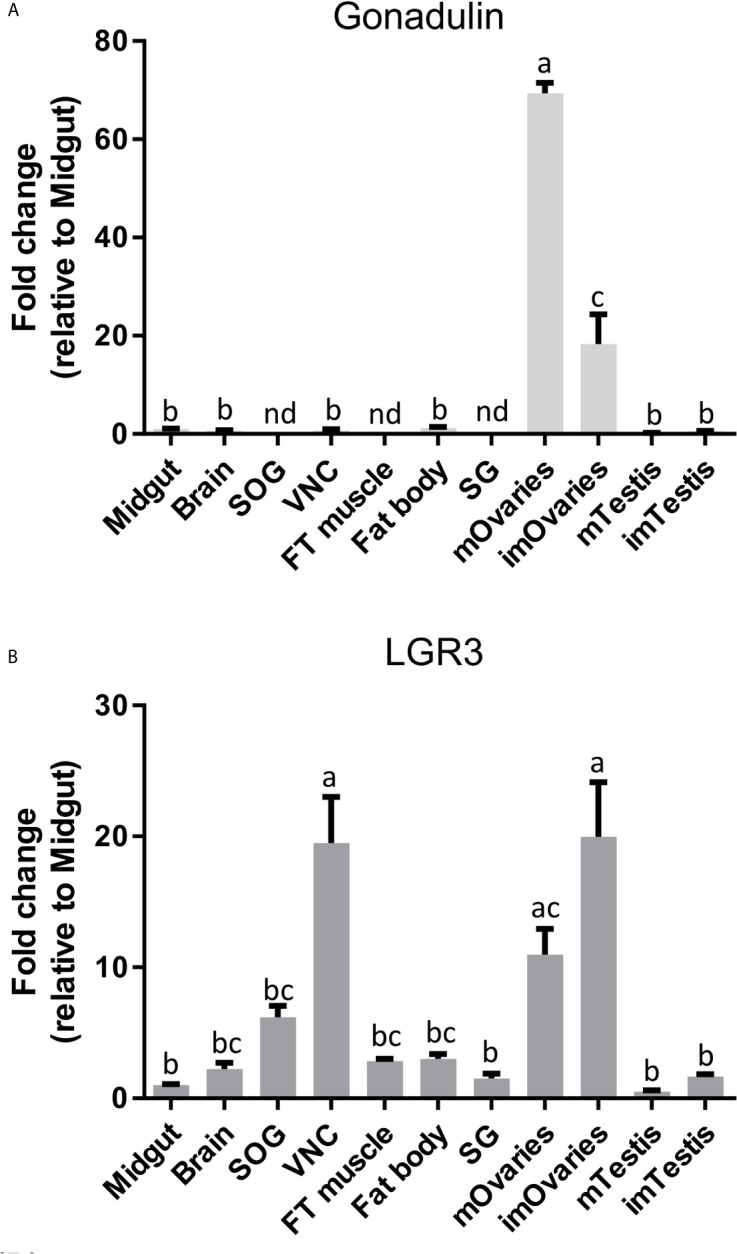
Distribution of gonadulin **(A)** and its putative receptor LGR3 **(B)** transcripts in different tissues from *L. migratoria* females, as well as testes from males. Midgut, brain, suboesophageal ganglion (SOG), flight muscle (FT muscle), salivary glands (SG), ventral nerve cord (VNC), fat body and ovaries (mOvaries) from 3-4-week-old sexually mature female, as well as testes from 3-4-week-old (mature tissue, mTestis), 1-2-days-old males (immature tissue, imTestis), and ovaries of 1-2-days-old females (immature ovaries, imOvaries) were included. The y-axis represents the fold change in expression relative to midgut (value ~ 1). The results are shown as mean ± SEM (n = 3, with each n composed of tissues from 2-3 insects). Significance of p < 0.05 is denoted using letters to indicate bars that are significantly different from others. nd, no detected.

Gonadulin sequences are highly variable (3, 19) and the two locust sequences are no exception, with numerous differences between them (**Figures 1A, B** and **Supplementary Figure S2**). The gonadulin gene is complete in the *L. migratoria* genome assembly and in the *S. gregaria* assembly it is present in two presumably adjoining scaffolds. Interestingly, the introns between the coding gonadulin exons are much smaller in the *L. migratoria* than in the *S. gregaria* gene. Furthermore, as in cockroaches, termites and stick insects (3), the gonadulin and aIGF genes are located tail to tail on the same scaffold, with their stop codons separated about 42,000 bp (**Figure 3**). Whereas aIGF is expressed in many tissues, the expression of gonadulin is much more limited as shown by both the number of gonadulin specific reads in the various SRAs (**Supplementary Spreadsheet**) and our RT-qPCR data (**Figure 6A**). The latter shows important expression in the ovary, both mature and immature, and very low levels of expression in the midgut, brain, ventral nerve cord, flight muscle, fat body, salivary gland and both mature and immature testis.

LGR3 has been shown to be the receptor for dilp 8 (20), and its orthologs therefore likely function as gonadulin receptors. We used RT-qPCR to study LGR3 expression and found it to be expressed in several tissues, but most abundantly in the ovary and the central nervous system (especially in the ventral nerve cord), but significant expression is also found in the fat body, testis, and flight muscles (**Figure 6B**).

**Figure 7:**
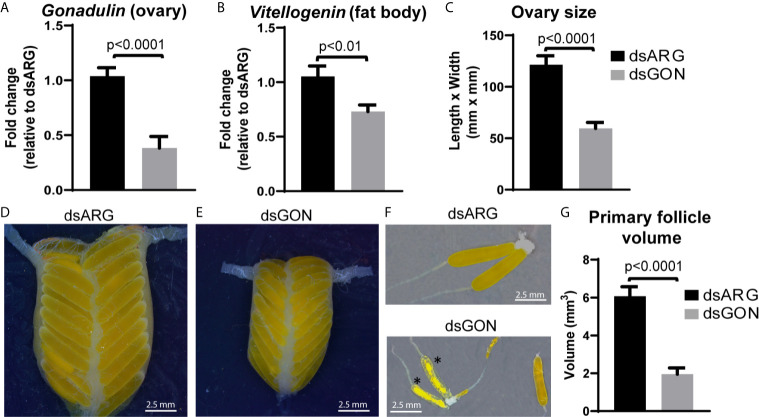
Effects of double stranded gonadulin RNA. Ovaries and fat bodies dissected from 10 day old adult virgin females injected with dsGON were dissected and the expression of gonadulin **(A)** and vitellogenin **(B)** was quantified relative to the expression of the same transcript in dsARG injected insects (n = 14-16). The y-axis represents the fold change in expression relative to dsARG (value ~ 1). **(C)** Size of ovary in dsARG and dsGON injected insects (n = 14-16). **(D, E)** Representative ovaries from adult females injected with dsARG **(D)** or dsGON **(E)**. **(F)** Representative ovarioles of females injected with dsARG or dsGON; **(G)** Volumes of primary follicles (means of 3 primary follicles from each ovary; n = 14-16). Statistically significant differences monitored using Student’s T-test. *, oocytes undergoing incipient resorption.

As gonadulin is almost exclusively expressed in the ovary, this suggests that it might constitute an important feedback signal from the ovary to the central nervous system and possibly other tissues, thereby acting as a hormone. We therefore used double stranded RNA to specifically inhibit gonadulin expression (dsGON). Animals were reinjected five days after the first injection and results analyzed five days later. Gonadulin transcript expression in the ovaries was strongly diminished (approx. 60%) in dsGON injected insects (**Figure 7A**). Visual inspection revealed ovaries and primary follicles that were much smaller in dsGON injected animals than in controls (**Figures 7C–G**) and the transcript expression of vitellogenin in the fat body was reduced (**Figure 7B**). When measured, the differences in size were statistically significant (n= 14-16, p < 0.001). Interestingly in some preparations there was evidence of incipient resorption in oocytes. This resorption was most evident in insects in which gonadulin expression was reduced to close to 100%. In those insects the effect on terminal follicle volume was also greater than those in which transcript expression was less reduced, suggesting a dose-dependency for gonadulin transcript knockdown and thereby gonadulin action (**Supplementary Figure S3**).

## Discussion

We describe two novel insulin-related peptides from migratory locusts, aIGF and gonadulin, a putative hormone that appears to be important for vitellogenesis.

The structure of locust aIGF looks particularly interesting. The first six of the ten Cys residues of aIGF are spaced like in insulin and may thus be expected to form a similar three-dimensional structure. Though it is impossible to be sure about the exact folding of locust aIGF, the first part of the molecule is clearly homologous to insulin and one might expect it to have a similar configuration, and the C-terminal tail to have its own three-dimensional structure imposed by Cys bridges. As there are neither apparent furin nor neuroendocrine convertase cleavage sites between the two parts, the final product is predicted to be a small protein that may on one side have an insulin-like structure that is expected to activate an RTK, and another piece of unknown function. As there are significant differences between the constitutive and regulated secretory pathways, one might *a priori* speculate that this second part of the molecule is necessary for successful processing and secretion. However, in locusts, the fat body also secretes IRP, which lacks such a C-terminal extension. Hence, the function of that second part of the molecule is most likely related to what happens after it has been released into the hemolymph. This second part might increase the half-life of the hormone, *e.g.* by facilitating its binding to a carrier protein. Alternatively, each of the two *L. migratoria* insulin RTKs (41) might preferentially bind to either aIGF or IRP.

In two insect species, aIGF seems to be important for growth of organs essential for successful sexual reproduction. Thus, in honeybees, aIGF stimulates development into a queen rather than a worker and its expression strongly increases when the diet consists of the protein-rich royal jelly (42–45). In the broad-horned flour beetle growth of its weapon is similarly dependent on aIGF from the fat body (5).

It is interesting to note that the tendency of a higher relative expression of aIGF as compared to IRP in solitarious phase locusts concords well with the rapid decrease observed in IRP expression on solitarization of 4^th^ instar *L. migratoria* and the reciprocal increase of IRP expression on gregarization (46). Egg laying in solitarious *L. migratoria* occurs much earlier than in gregarious locusts (47) and these data would thus be compatible with the hypothesis that *L. migratoria* is another species in which aIGF stimulates sexual maturation. Although this hypothesis needs to be confirmed by experimental data, it suggests that the aIGF/IRP expression ratio could be an important factor in phase polymorphism. In *R. prolixus* an aIGF is expressed in the ovaries (48) and was reported to influence growth (9). In addition, in *B. germanica* the expression of an aIGF-like transcript has a profile parallel to vitellogenin transcript expression in the fat body (6) indicating that aIGF is associated not only with sexual maturation but also with reproduction.

Gonadulin is the second novel locust ILP identified here. This putative hormone is predicted to have the same disulfide bridges as insulin given the spacing between the six Cys residues in its precursor. However, its primary amino acid sequence is so different from other insect ILPs, it is difficult to find by using the BLAST program or the sratoolkit. As gonadulin’s expression in *L. migratoria* appears to be largely limited to the ovary, it is almost certainly secreted through the constitutive secretory pathway. In the regulated secretory pathway insulin precursors are typically cleaved in the secretory granules by one or two neuroendocrine convertases that remove the peptide connecting the A- and B-chains. These enzymes are generally absent from the constitutive pathway. Furin could potentially cleave some sites, but its specific requirements for processing are not well known, making it impossible to predict the processed structure of gonadulin and it is therefore not clear whether the mature peptide is a single-chain or a two-chain molecule.

Inhibition of gonadulin expression in the ovary led to much smaller ovaries and primary follicles as well as a decrease in the amount of vitellogenin mRNA in the fat body. The effects that we observed with dsGON are remarkably like those observed after RNAi inhibition of its putative receptor LGR3, previously incorrectly identified as LGR4 (38). Thus, knocking down LGR3 in *L. migratoria* resulted in phenotypes with defects in vitellogenin accumulation in the developing oocytes, blocked ovarian development, and impaired oocyte maturation (38). The observed effect of knocking down the gonadulin-signaling pathway on vitellogenin production, both in this study and the study of Zheng et al. (38), could be due to an effect of gonadulin signaling on the fat body, central nervous system, or both. Although the specific mechanism leading to juvenile hormone (JH) signaling for vitellogenin synthesis by the fat body has been extensively studied in *L. migratoria* (49–51), the expression of LGR3 in the fat body along with results obtained from RNAi experiments suggest that gonadulin signaling may act, *via* interaction with other pathways, on the production and translation of vitellogenin mRNA.

In our experiments, primary oocytes undergoing resorption were more often observed in dsGON treated insects. Among several factors known to trigger oocyte resorption in insects, nutritional deficiency is the one that is most likely applicable in this case (52). As the insects were fed in identical fashion as controls, this suggests that they consumed less food. The expression of LGR3 in the central nervous system may thus be consistent with gonadulin signaling a need for more food. It may also indirectly exert its effect on vitellogenin synthesis by controlling the production or release of other insect hormones, as was proposed for *D. melanogaster* (19), while its expression in the ovary itself may reflect an autocrine stimulation of vitellogenin uptake.

Whatever, its exact mode of action, our results point to a powerful role of gonadulin in reproduction. It is possible that gonadulin is a feedback signal from the ovary in the central nervous system - fat body - ovary axis to ensure successful reproduction. However, if this were the case, the apparent absence of gonadulin in many insect species clearly shows that such a function is not an ubiquitous one. As insulin and related peptides are evolutionary conserved sensors of the nutritional status of organisms and involved mainly in the control of anabolic processes, the expression of the putative receptor to gonadulin in several tissues might indicate a need for resources, perhaps specifically amino acids that are greatly needed during vitellogenesis.

## Data Availability Statement

The raw data supporting the conclusions of this article will be made available by the authors, without undue reservation.

## Author Contributions

JV, JL, AL and IO designed the experiments and mapped out the manuscript. JV performed *in silico* analysis and JL performed the experiments. JV and JL wrote the initial draft of the manuscript and prepared all the figures. AL and IO reviewed and contributed to the writing of the final manuscript. All authors contributed to the article and approved the submitted version.

## Funding

This work was supported by the Natural Sciences and Engineering Research Council of Canada Discovery grants to AL (RGPIN-2019-05775) and IO (RGPIN-2017-06402).

## Conflict of Interest

The authors declare that the research was conducted in the absence of any commercial or financial relationships that could be construed as a potential conflict of interest.

## Abbreviations

aIGF, arthropod insulin-like growth factor; ILPs, insulin-like peptides; IRP, insulin-related peptide; RTK, receptor tyrosine kinase; LGR, leucine rich repeat G-protein coupled receptor; PBS, phosphate-buffered saline; RT-qPCR, reverse transcription/quantitative PCR; RNAi, RNA interference; dsRNA, double-stranded RNA; cDNA, complementary DNA; SRA, short read archives; Vg, vitellogenin, dsGON, gonadulin dsRNA, GSP, gene specific primers, dsARG, ampicilin resistance gene dsRNA.
