# Supplementary material for: Identification of Gonadulin and Insulin-Like Growth Factor From Migratory Locusts and Their Importance in Reproduction in Locusta migratoria
Source: Front Endocrinol (Lausanne). 2021 Jun 4;12:693068. doi: 10.3389/fendo.2021.693068 (PMC8220825; doi:10.3389/fendo.2021.693068)
Supplement: Supplementary file 1 [file DataSheet_1.pdf]

Supplementary data for:

Identification of gonadulin and insulin-like growth factor from migratory locusts and the importance of gonadulin for vitellogenesis in *Locusta migratoria*

Contents:

Sequence alignment of aIGFs from Hemimetabola page 2

Sequence alignment of gonadulins from Hemimetabola page 3

Coding and amino acid sequence for *Locusta* aIGF page 4

Coding and amino acid sequence for *Schistocerca* aIGF page 4

Coding and amino acid sequence for *Locusta* gonadulin page 5

Coding and amino acid sequence for *Schistocerca* gonadulin page 5

Coding and amino acid sequence for *Locusta* LGR3 page 6

Coding and amino acid sequence for *Schistocerca* LGR3 page 8

Coding and amino acid sequence for *Locusta* LGR5a page 10

Coding and amino acid sequence for *Locusta* LGR5b page 13

Coding and amino acid sequence for *Schistocerca* LGR5a page 16

Coding and amino acid sequence for *Schistocerca* LGR5b page 19

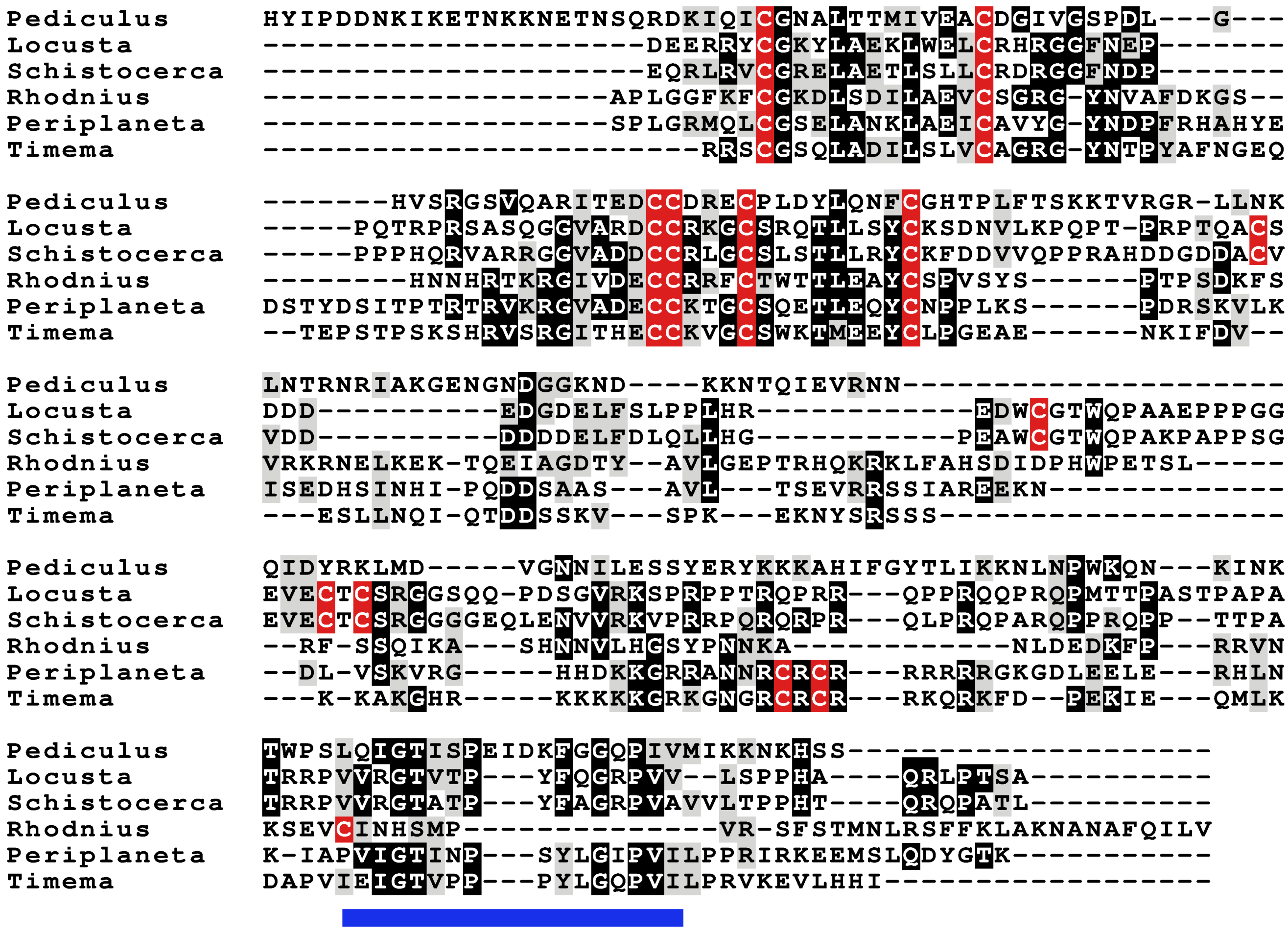

Fig. S1. Sequence alignment of locust aIGFs and long isoform aIGFs from a few other Hemimetabola. Note that they have an N-terminal insulin-like structure followed by a C-terminal tail that has two or four additional cysteine residues. Cysteine residues are indicated in red and other conserved amino acid residues are highlighted in black and conserved substitutions in grey. Underlined in blue is a sequence that was previously identified as partially conserved in insect aIGFs. Sequences described here on page 4 of these supplementary data and from Veenstra (2020b).

```
Rhodnius      ---SPCDKQYIKYLTLYSCTYPKRKISLFDILHLPLKEVNKRFETLLGKNSAKMF
Timema        ----ECDKARIMNYIMYSCSKKKRSPAD--HLIYP-------------------S
Locusta       ---NTCDGERILELMKNACVVRRRRAAAPEARQAV--------------------
Schistocerca  ---STCDGESILKLMRDACVVRRRRDASPGERAAG--------------------
Periplaneta   EKYENCSKKL-RQLILDSCNEPKNKRSA---------------------------
Pediculus     -----CDTKWIRLVYMMSCGKKKRDSSTSSFNETLLR------------------

Rhodnius      ENWELPNWNNTIKNTTNSKFEIERLKTILLRKNNRQKRKAQSGGYLHLGQMAKDF
Timema        ENWHLD-----LPDAPSH--NGGKLSEMLVVAMNPSH--PVLYEWMMEGEEPTIF
Locusta       ---------------------HDQISQ-LRAQRLPQDLDDQFWDGLQELA-----
Schistocerca  ---------------------EEQLVL-PRAGRLPHGLHDQFWESLLELA-----
Periplaneta   ------------------VFER-HGFN-MHSPHLPQDRSQQVTSSVLLGKILGVP
Pediculus     -------------NMPSDFYGAVKYLK-ENDPQNPMFKDDQFLYRILEDGIHGFP

Rhodnius      DYWHFM-------QDETPFKATELMKLMWQCCTSD--IPCYVSNFSRIC------
Timema        DDLSKK-------TRSIFVYPEKAQRVIEQCCDKE--QYCDVNTFLGACK-----
Locusta       ------GSEQKTVLARALRKSSKFHQLITACCR----RACTAKDFRLLCGSPRKP
Schistocerca  ------DSEEKTVLSRALRKSSKFHQLISVCCR----RTCTAKDFRVLCGPPRKT
Periplaneta   SQWTEEELSSHQINKQFRRNNQSVRNLIIECCV----DGCTPNQIMGLCD-----
Pediculus     DQWEVDEINDDKINENHRRSS-KIDKLIEECCKVPPVRTCSEKTFKGAC------
```

Fig. S2. Sequence alignment of locust gonadulin and those from a few other Hemimetabola. Cysteine residues are indicated in red and other conserved amino acid residues are highlighted in black and conserved substitutions in grey. Sequences described here on page 5 of these supplementary data and from Veenstra (2020b).

Locusta migratoria aIGF coding sequence

ATGCGCGTGCTGCTGGCGGTGACCGTCTTCTGCGCATGCGCCGTGTGGCTGCCCGCAGCG
GCGGACGAGGAGAGGCGTTACTGCGGCAAATACCTGGCGGAGAAGCTGTGGGAACTGTGC
CGGCACCGCGGGGGCTTCAACGAGCCGCCGCAGACCCGCCCGCGGTCAGCGAGCCAAGGC
GGCGTGGCCCGCGACTGCTGCCGGAAAGGCTGCTCGCGGCAGACGCTGCTCAGCTACTGC
AAGTCCGACAACGTGCTGAAGCCGCAGCCGCCGCCGCCGCGCCCCACCGAGGCGTGCAGC
GACGACGACGAGGACGGAGACGAGCTGTTCAGCCTGCCGCCGCTGCACAGGGAGGACTGG
TGCGGCACGTGGCAGCCTGCAGCGGAGCCGCCTCCTGGTGGTGAAGTGGAGTGCACGTGT
TCCAGAGGTGGCGGCCAGCAGCCGGATAGCGCGGTGCGCAAGAGCCCGCAGCCGCCGACT
CGTCAGCCTCGTCGCCAGCCGCCTCGTCAGCCGCCGACGACGCCCGCCTCGACGCCCGCG
CCCGCGACGCGGCGCCCCGTCGTCAGAGGCACGGTGACGCCCTACTTCCAGGGCCGACCG
GTCGTCCTATCCCCCCCTCACGCGCAGCGCCTGCCGACCTCCGCCTAG

Nucleotides highlighted in yellow correpond to primers used for transcript quantification.

Locusta migratoria aIGF

MRVLLAVTVFCACAVWLPAAADEERRYCGKYLAEKLWELCRHRGGFNEPPQTRPRSASQG
GVARDCCRKGCSRQTLLSYCKSDNVLKPQPPPPRPTEACSDDDEDGDELFSLPPLHREDW
CGTWQPAAEPPPGGEVECTCSRGGGQQPDSAVRKSPQPPTRQPRRQPPRQPPTTPASTPA
PATRRPVVRGTVTPYFQGRPVVLSPPHAQRLPTSA*

Schistocerca gregaria aIGF coding sequence

ATGCGGCCGCTGCTGGCCGTGTTGTGCGTGGCCTGCGCCGGTGCCTTCCTCTGTCCCCGG
GCGGCGGCCGAGCAGAGGCTGCGCGTCTGCGGCCGAGAGCTGGCCGAGACGCTGAGCCTG
CTCTGCAGGGACCGCGGAGGCTTCAACGACCCACCTCCGCCGCACCAGCGTGTGGCACGG
CGCGGCGGCGTGGCCGACGACTGCTGCCGGCTGGGCTGCTCGCTCAGCACGCTGCTGCGC
TACTGCAAGTTCGACGACGTGGTGCAGCCGCCGCGGGCACACGACGACGGCGACGACGCC
TGCGTCGTCGACGACGACGACGACGACGAGTTGTTCAGCCTGCAGCTCCTGCACGGGCCG
GAGGCGTGGTGCGGTACGTGGCAGCCGGCGAAGCCGGCGCCGCCTAGTGGTGAAGTGGAG
TGCACGTGTTCCAGAGGCGGCGGCGGCGAGCAGCTGGAGAACGTGGTGCGCAAGGTCCCG
CGGAGGCCTCAGCGCCAGCGCCCTCGTCAGCTGCCTCGTCAGCCAGCTCGTCAGCCGCCT
CGTCAGCCGCCGACGACCCCCGCGACTCGGCGCCCCGTCGTCAGAGGCACGGCGACGCCC
TACTTCGCGGGCCGGCCCGTCGCCGTCGTCCTCACCCCTCCGCACACTCAGCGCCAGCCA
GCAACACTGTAG

Schistocerca gregaria aIGF

MRPLLAVLCVACAGAFLCPRAAAEQRLRVCGRELAETLSLLCRDRGGFNDPPPPHQRVAR
RGGVADDCCRLGCSLSTLLRYCKFDDVVQPPRAHDDGDDACVVDDDDDDELFSLQLLHGP
EAWCGTWQPAKPAPPSGEVECTCSRGGGGEQLENVVRKVPRRPQRQRPRQLPRQPARQPP
RQPPTTPATRRPVVRGTATPYFAGRPVAVVLTPPHTQRQPATL*

Locusta migratoria gonadulin coding sequence

ATGCGCGCCCGGCTGACCGGCATTCGACTCGCAGTCGGCAACATGCAGCTCTACGTGGCA
GTCCTGGTGTTCGTGGGCGCGCTGTGCGCGTCGGCGGCGGCCAACACGTGCGACGGGGAG
CGCATCCTGGAGCTGATGAAGAACGCCTGCGTGGTGCGGCGCAAGCGCGAGGCGTCGCCA
GCGGCGCGACAGGCCGTCCACGACCAGATCTCCCAGCTGAGGGCGCAGCGCCTGCCCCAG
GACCTGGACGACCAGTTCTGGGACGGGCTGCAGGAGCTGGCGGGCTCGGAGCAGAAGACG
GTGCTGGCGCGCGCGCTGCGCAAGAGCTCCAAGTTCCACCAGCTGATCACCGCCTGCTGC
CGCAGGGCCTGCACCGCCAAGGACTTCCGCCTGCTCTGCGGCTCCCCGCGCAAGCCCTGA

Nucleotides highlighted in yellow correpond to primers used for transcript quantification.
Nucleotides in red indicate location of primers used for making dsGON.

Locusta migratoria gonadulin

MRARLTGIRLAVGNMQLYVAVLVFVGALCASAAANTCDGERILELMKNACVVRRKREASP
AARQAVHDQISQLRAQRLPQDLDDQFWDGLQELAGSEQKTVLARALRKSSKFHQLITACC
RRACTAKDFRLLCGSPRKP*

Schistocerca gregaria gonadulin coding sequence

ATGTTCGGCTCGCGGTGCAGCACGCATTACGCAGTCGGCGACATGCAGCTCCACGTGGCA
GTCCTGGTGTGTGTCGGCGTGCTGTGCGCCACGGCGGCGGCCAGCACGTGCGACGGCGAG
AGCATCCTGAAGCTGATGAGGGACGCGTGCGTGGTACGCCGGAGGCGCGACGCCTCGCCC
GGAGAGCGGGCGGCGGGGGAGGAGCAGCTTGTGCTGCCGCGCGCCGGGCGCCTGCCGCAC
GGCCTGCACGACCAGTTCTGGGAGAGCCTGCTGGAGCTGGCGGACTCCGAGGAGAAAACG
GTGCTGTCGCGCGCGCTGCGCAAGAGCTCCAAGTTCCACCAGCTGATCAGCGTCTGCTGC
CGGCGAACCTGCACCGCCAAGGACTTCCGCGTGCTGTGTGGGCCTCCACGCAAGACGTGA

Schistocerca gregaria gonadulin

MFGSRCSTHYAVGDMQLHVAVLVCVGVLCATAAASTCDGESILKLMRDACVVRRRRDASP
GERAAGEEQLVLPRAGRLPHGLHDQFWESLLELADSEEKTVLSRALRKSSKFHQLISVCC
RRTCTAKDFRVLCGPPRKT*

>*Locusta migratoria* LGR3 coding sequence

ATGCGGTGCGGCCGGGGCGCGCTGTGCGGGGCCGCCGCCGCCGCCTGCCTGCTCGCC
GCCGCGCTGCTCTGGATCCTCGCGCAAGGAAGCTGCTCGCGGGGGAGCTTCGCCTGCACC
AACTCGTCGCTGTGCATCCCGCAGCACTACGCGTGCAACGGACACGCCGACTGCCCTGAC
GCCAGCGACGAGGACGTCTTCGTTTGTGCCGACTTCAACGGCTACTCGTTCGTGAACGAG
ATGGTGGACAACGCGAGCGGCGCGGCGTCTGTGGTGGGGGAGGACCAGGAGGAGGAGGAG
GAAGAGCGGGAGGAGGCGTGCGGCGCGCCGGCCGCCTGCTGGTGCCGCGGCCAAACCCTG
CGCTGCTCCGGGGAGCGCCTCACCGCCGTGCCACACGCACTCTCCACGCAGCACACCAAC
GTGATCCTGAACAACAACTCAATACACGTGATCGCTGCTGATGCCTTCAGTAATTATTCA
TTAACCATCCTGAACTTGGATGACAATGGTCTCGAGTTTCTTCCACATGGACTCTTCGAT
GGACAGACACAACTGGAAAGATTGTACCTGTCGGACAACGCGCTGGGCGGGGACAGCGTG
TCTCGGCTGTCTGGGCTGGGCGCGCTGCTCTGGCTGTTCCTGGACGGCAACCGCCTGCAC
CACGTCCCGCTAAGTCATCTGGCCGGCCTGCGCAGCCTCATCTGGCTGGACTTATCACGG
AACGCGCTGACGTTGGAAGGTGAACAATTCCCAGAACTTCCTCGACTTAAACTTCTAATG
TTGCAGGAGAATAATATCGAGTATATTGGTGAAAATCTGTTTGCCGGATTAAGAGGTCTT
ACGCAATTGAACCTAAGAAGAAATGCCATCCGATCGATAGATGTCAATGCTTTTAGGAAC
CAGAAACATCTGGAGCACTTGGACATATCAGAGAACCAGCTGACGACGCTGCAGAACAAA
GTCTTCTCACCACTGCACGCCCTCAGCAGACTGTCGCTTGGAAACAATCCAGTCGCCTTC
ATACCAGGAGATTTATTTCAGAACTTGACAAATCTTCAGTCACTAGACCTCAGAGCCATC
AACATAGAGAACATAGACACCACGATGTTCACGCTACTGAAGAAGTTAGAGTTTGTGTAT
TTCAAGACATTCAATTACTGTAAATATGCTCCGACTGTTCCCAAATGCAAGCCTAATACA
GACGGCGTGTCGTCGTCTGAGCACCTGCTGTCTCGGCCGGAGCTGCGCGCGTCGCTGTGG
GCGACCGCCGGCTGCACGGCGCTGGGCAACGCGCTGGTGCTGCTGGGCCGCGGACGCGCC
GCCGCCGTCGCCACCGACAACCGCGTCGTCAGCCTCGTCGTCCGCAACCTCGCAGCGTCG
GACCTGCTGATGTCGGTGTACCTGGCGGTGATCGGGTGGCAGGACGTGCAGCTGCGGGGC
GTCTACCACCGCGCCGCGCACTCCTGGACCACCTCGTGGCTCTGCACGCTCGCCGGCATG
CTCGCCATGACCTCCTCAGAGGTGTCGGTGCTGATCATGGTGTTCATGTCGGTGGAGCGG
TTCTCGCTGATCGCGCTGCCGCTGTCGCACAGCGCCAGCCTGGCCGTGCGCAGCGCCGCC
ACCGCGCTCGCCCTCATCTGGCTCGCCGGCATCAGCCTCGCCGTCATACCAGCAATACAC
TACCGCGGCTCTGCAAGATACTACGGTATAAACAGCATGTGCTTCCCTCTGCACATTGAC
GACCCTTTCTTCCACGGCTGGCAGTATTCCGCATTTATATTATTTGGAGTCAACATGACG
GGGCTGGTGGTGATGGCGGGCCTGTACACGGGCATGCTGGTGAGCATCGTGCGCACGCGG
CAGGCGACGCCCGCGTCCCTGCGCGGGGAGCTGGAGTTCGCGGCGCGCTTCTTCTTCATC
GTGGTGACCGACGCCGCCTGCTGGGCGCCGCTCATCACATTCCGCGCCCTCGCCATGTTC
AGCTTCCACATTCCCAGCGAGGTGTACGCGTGGCTGGTGGTGCTGGTGCTGCCGGTGAAC
TCGGCGGTGAACCCCGCGCTGTACACGTTCACGACGCCGCGCTACTGGGCGCGCCTCTGC
CGCCGCCGCCGCCGCAGCAGCCAGCCGGCCGCGCTGCCCAGGCACCAGTACGCGCCCTGC
TCGCGCCTCTCCTCGCTGCCGCTCGTCAAGGTCATGAACGAGGCAAAATGCTGA

Nucleotides highlighted in yellow correpond to primers used for transcript quantification.

>*Locusta migratoria* LGR3

MRCGRGALCGAAAAAACLLAAALLWILAQGSCSRGSFACTNSSLCIPQHYACNGHADCPD
ASDEDVFVCADFNGYSFVNEMVDNASGAASVVGEDQEEEEEEREEACGAPAACWCRGQTL
RCSGERLTAVPHALSTQHTNVILNNNSIHVIAADAFSNYSLTILNLDDNGLEFLPHGLFD
GQTQLERLYLSDNALGGDSVSRLSGLGALLWLFLDGNRLHHVPLSHLAGLRSLIWLDLSR
NALTLEGEQFPELPRLKLLMLQENNIEYIGENLFAGLRGLTQLNLRRNAIRSIDVNAFRN
QKHLEHLDISENQLTTLQNKVFSPLHALSRLSLGNNPVAFIPGDLFQNLTNLQSLDLRAI
NIENIDTTMFTLLKKLEFVYFKTFNYCKYAPTVPKCKPNTDGVSSSEHLLSRPELRASLW
ATAGCTALGNALVLLGRGRAAAVATDNRVVSLVVRNLAASDLLMSVYLAVIGWQDVQLRG
VYHRAAHSWTTSWLCTLAGMLAMTSSEVSVLIMVFMSVERFSLIALPLSHSASLAVRSAA
TALALIWLAGISLAVIPAIHYRGSARYYGINSMCFPLHIDDPFFHGWQYSAFILFGVNMT
GLVVMAGLYTGMLVSIVRTRQATPASLRGELEFAARFFFIVVTDAACWAPLITFRALAMF
SFHIPSEVYAWLVVLVLPVNSAVNPALYTFTTPRYWARLCRRRRRSSQPAALPRHQYAPC
SRLSSLPLVKVMNEAKC*

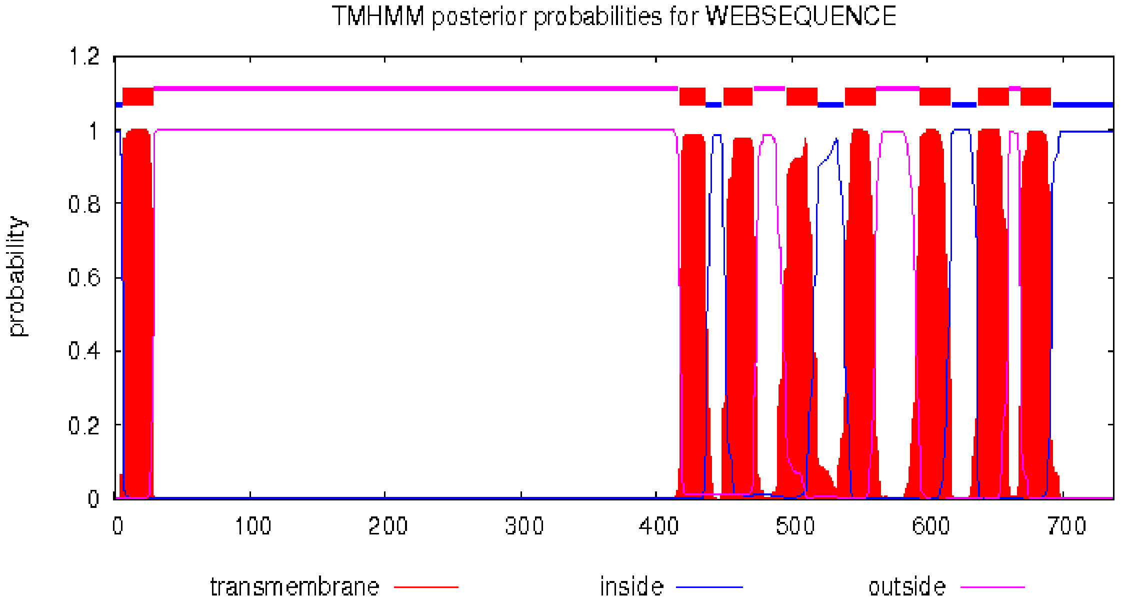

Prediction of Transmembrane regions of *Locusta migratoria* LGR3 as determined at:
http://www.cbs.dtu.dk/services/TMHMM/

>*Schistocerca gregaria* LGR3 coding sequence

ATGCGCTGCGGCCGAGGCGCGCTGTGCGGCGCGGCGGCCGCTGCCTGCGCGCTGCTGCTC
GCCACGCTGCTCTGGATCCTCGCCCAAGGCAGCTGCTCCCGGGCCAGCTTCGCCTGCAGC
AACTCGTCGCTGTGCATCCCGCAGCGTTACATGTGTAACGGACACGCAGACTGCCCAGAT
GCCAGCGATGAAGACGTCTACGCTTGTGCTGACTTGAACGGCTACTCGTTCGTGAACGAG
ATGGTGGGGAACGCGAGTGGTGCTGCGGTTGTCCTGGAGGCGGAAGGCGAGGACGAGGAA
GAGGAAAACGAGGAGTCTTGCGGCGCGCGGGCCGCCTGCTGGTGCCTCGGGTCCGCGCTG
CGCTGCGCTGGCGTCGGGCTGTCCGCCGTGCCTACCGGCTTGTCACCAGACCTCACTCAC
ATGGTGCTGAACAACAATTCAATCCAAGTGATCGCTGCTGACGCCTTCAGTAATTATTCA
TTAAACGTCCTGAACCTGGATGAAAATGGTCTTGAGTTTCTTCCACCTGGACTATTCGAT
GGGCAGACACAACTGGAAAGATTGTACCTGTCGGACAACGCGCTGCGGGCCGACAGCGTG
CCGCAGCTGTCGCTGCTGTCCTCGCTGCTGTGGCTCTTCCTGGACGGCAACCGCCTGCAC
CACGTTCGCCTGCAGCACTTGGCCGGCCTGCGCAGCCTCGTCTGGTTGGACTTGTCAAGG
AATGCGCTCACGTTGGAAGGTGAGAAATTTCCCGAGCTACCTCGACTGAAACTTCTAATG
CTGCAGGAGAATAACATAGAGCTGATTGGGGAGGATCTGCTTGCCGCGTTAAGAGGCCTT
ACACAATTGAACCTGAGAAGAAATGCCATCCGATCAATAGATGTTAATGCGTTTAGGAAC
CAGAAACATCTGGAGCACTTAGATATATCAGAGAACCAGCTCAAGACGCTACAGAACAGG
GTGTTCTTACCACTCGGGACCCTCAGCAGATTGTCCCTTGGAAATAATCCAGTTACCTTC
ATACCAGGAGATTTATTCCAAAACTTGACAAATCTTCAGTCATTAGACCTTCGGGCCATC
AACATAGAGAACATAGACACCACGATGTTCACGTTACTGAAGAAATTAGATTTTGTGTAT
TTCAAGACGTTCAATTACTGTAAATATGCTCCGACTGTCCCAAAATGCAAGCCGAGTACA
GACGGCGTGTCGTCGGCGGAGCACCTGCTGTCGCGGCCGGAGCTGCGCGCTTCTCTGTGG
GCGACGGCGGGCTGCACGGCGGTGGGCAACGCGCTCGTGCTGCTGGGCCGCGGGCAGGCC
GCCAGCGCCGCCGACAACCGCGTCGTCAGCCTCGTCGTCCGCAACCTCGCAGCTTCTGAC
TTGCTGATGTCGGTGTACCTGGCGGTGATTGGGTGGCAGGACGTGCAGCTGCGGGGCGTC
TACCACCGCGCTGCGCACTCCTGGACCACCTCCTGGCTCTGCACTCTCTCCGGCATGCTC
GCCATGACGTCCTCAGAGGTTTCAGTGCTGATAATGGTGTTCATGTCGCTGGAGCGGTTC
ATTCTGATCGCGCTGCCGCTGTCTCACAGCACCAGCTTGGCCGTGAGGAGCGCCGCTGCC
ACGCTGGCCCTCATCTGGATCGCAGGCATCAGTTTGGCTATCATTCCAGCAATACACTAT
CGCGGATCGACAAGATACTATGGCATAAACAGCATGTGCTTTCCTCTGCATATTGACGAT
CCTTTCTTCCTTGGCTGGCAGTATTCTGCATTTATATTATTTGGGATCAACATGACAGGG
TTGGTGGTGATGGCAGCGCTGTACACAGGCATGTTCATAAGCATCGTACGAACGCGCCAC
GCAACGCCCGTCTCGATGCAGGGTGAGCTGGAGTTCGCGGCTCGCTTCTTCTTCATTGTG
GTCACTGACGCCGCCTGCTGGGCGCCACTCATCACTTTCAGAGCCCTCGCCATGTTCAGC
TTCCACATTCCCAGTTACGAGGTGTACGCGTGGCTGGTGGTGCTGGTGCTGCCGGTGAAC
TCTGCGGTGAACCCGGCGCTGTACACGTTCACCACGCCGCGCTACTGGGCGCGCCTGTGC
GGGCTGGTGTGCGACCGTCGCAGCCGCCGCCGCAGCCCCGAGACCCAGGGACTCAGTCGC
CAGCAGTACGCACAGTGCTCGCATCTCACGTCGCTGCCGCTCGTCAAAGTAAGCACCACA
CAACTTCTTCTCTTTTGTAATATTGTTGCAGAAGCATAA

>*Schistocerca gregaria* LGR3

```
MRCGRGALCGAAAAACALLLATLLWILAQGSCSRASFACSNSSLCIPQRYMCNGHADCPD
ASDEDVYACADLNGYSFVNEMVGNASGAAVVLEAEGEDEEEENEESCGARAACWCLGSAL
RCAGVGLSAVPTGLSPDLTHMVLNNNSIQVIAADAFSNYSLNVLNLDENGLEFLPPGLFD
GQTQLERLYLSDNALRADSVPQLSLLSSLLWLFLDGNRLHHVRLQHLAGLRSLVWLDLSR
NALTLEGEKFPELPRLKLLMLQENNIELIGEDLLAALRGLTQLNLRRNAIRSIDVNAFRN
QKHLEHLDISENQLKTLQNRVFLPLGTLSRLSLGNNPVTFIPGDLFQNLTNLQSLDLRAI
NIENIDTTMFTLLKKLDFVYFKTFNYCKYAPTVPKCKPSTDGVSSAEHLLSRPELRASLW
ATAGCTAVGNALVLLGRGQAASAADNRVVSLVVRNLAASDLLMSVYLAVIGWQDVQLRGV
YHRAAHSWTTSWLCTLSGMLAMTSSEVSVLIMVFMSLERFILIALPLSHSTSLAVRSAAA
TLALIWIAGISLAIIPAIHYRGSTRYYGINSMCFPLHIDDPFFLGWQYSAFILFGINMTG
LVVMAALYTGMFISIVRTRHATPVSMQGELEFAARFFFIVVTDAACWAPLITFRALAMFS
FHIPSYEVYAWLVVLVLPVNSAVNPALYTFTTPRYWARLCGLVCDRRSRRRSPETQGLSR
QQYAQCSHLTSLPLVKVSTTQLLLFCNIVAEA*
```

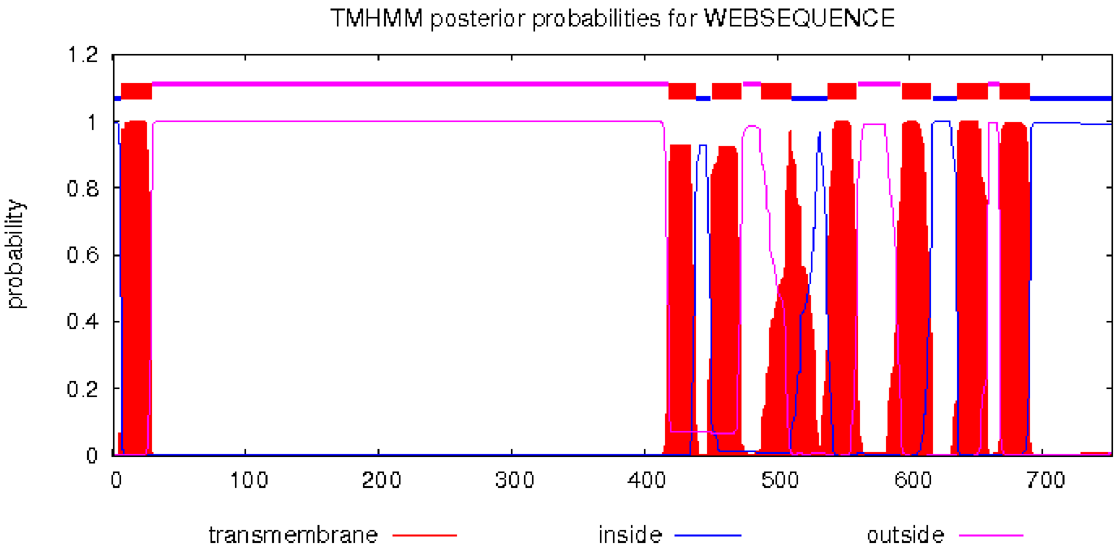

Transmembrane regions of *Schistocerca gregaria* LGR3 as determined at:
http://www.cbs.dtu.dk/services/TMHMM/

>*Locusta migratoria LGR5a* coding sequence

ATGCCTCATTACTGCAGGACCGTTTGCCTTTGTCTGCTGATGCTCTTGTTGAGCGCACCT
TTCTCCGAAGCCGCTGAAATCGAAAGAGAGTCGACGGCAGCAGAGCCGTCCCGCTGGCAG
TGCAGCAGCACGCAGGAGCAGCCCGGGCGGCAGGAGGTGAGCTGCGGCGACGGCTGGTGC
GTGGCCGAGGGCGCTCTCTGCAACGGGGTGCCCGACTGCGCCGGCGGCCAGGACGAGGCC
GTCCACCACTGCGGTTGCCTTCCAAATGAATTCCGGTGCAGTAATTCGTGCATCGACCTG
GTGAGGCGCTGCGATCGCGAGGTCGACTGCCCGACCGGCGAAGACGAAGACGACTGTGAG
TCGTTCGTGTGCCCAGTGACCCACTTCAAGTGCGCCAACCACTTCTGCGTGCCCGAGGAC
GCTGTCTGCAACTTCGAGGACGACTGCGGCGACGGGTCGGACGAGCTAAGCTGCTCGCAT
CGATCTTGCTGGAATTCAGAGTTCCGGTGCGACAACGGAGAGTGCATGCGTCCTGGCTTC
GTATGTGACGGCATCGTCGACTGCAAGGACGCCAGCGACGAAGCTTATTGCTCACCAGCC
GAATTTCGCCTCTGCGGAGACGGAACGCGCGTCCACAAGTTTTTTGGTGTGACGGGTGG
CCACACTGCCACGACAACCACGCCGATGAACTCAATTGCAGTGAGTGCAATGGAGCGGGC
GAGTACCTCTGCCCGAATGGTCGCTGCATCAGGCGTGCCAACGTCTGCGACTCATACTGC
GACTGCGTGCCCACTGCCAGAGGGCAACCGTGTGCGGACGAGGTGGACTGTGGCAGCAAA
TACGTCAAGCAGCACGGTGTAAGTCTCTGCATACCGGGTGAAACATTATCATGTTTTCTG
CCGTCCACCAAGAAGTACCGAGACGAACGTTGCATCTCATCAGACTTCATATGTGACGGC
TACAATGATTGTCACAACGGTAGATTCATCTCCGATGAATTTGGCTGTGACATGCTTACA
TCCAGCTACCCAGAAGATGTGTTCAACTGTTCTGAGGGTCGAGGTATTCCTGTGTCACTT
GTGTGTGATTTCAAGCGAGACTGCCTGAATGGAGAAGATGAACAGTTCTGTGGAAAAAGA
GATTGCTCAGTAAAAGAATACACTTGTGCCAGTGGGCAATGTATTCCATTGGAACAGGTC
TGCGACCTCAATTTTGATTGTTGGGATAAGACTGATGAAATGAATTGCTCAAATCTGAGG
TGTTCTGAAGGGTACCAGAGATGTAGATATAGTGGTCAATGTATTCCTGCAGCTCGATGG
TGTGATTACAGTGTGGACTGCTTTGATGGCAGCGATGAAGAGGAATGTGAATCTGTTCAG
CGAGATTGCCGATCTGATGAATTTCGCTGCAATAGTGGTCAGTGCATATTTGCTGACCTA
CAGTGCTTCAAGCCTGGTACTGAACACACTGCATGTGCTGATGGATCACATCTAATTAAC
TGTGATAATTGGACATGTCCTGACGGGACTTTCAAATGTAGAAATGGTCCTTGCCTGAAT
ATGTCATTATTGTGCAATAAGAAGATTGACTGCAAAGATACTTGGGTGGATGAAGATCAT
TGTCCATTTGAGTGCTGTGGTGACAATCGCTGCTTCTGTCTGGATACAAAAATTAACTGC
AGTAGCCTTGAGCTGAGGGATCTCCCATATGGAATTGAAGTTCAAATAAATCGCTTCTAC
ATGGCCAATAACTTGCTGAACTATTCACTCAATGTTGATACCTTCTCAAAACTAGACCGG
CTGTTATACTTGGATCTGACAAACAACAGTATTTCATGGCTACCTCCACTGATGTTTCGT
AATCTATGGCGACTGCACATCCTAAACTTGCAGAATAACAAGCTATCTGTAATTAGCAAT
GGTACATTCTTTGGATTACCCAACCTTGGAACACTTCATCTCCATGGTAACAACATTCAG
AGCTTAGAGCCAATGGGCTTCTATGGTCTCTCTTCACTGAAAGCACTTGATCTTAGACAT
CAAAGGATAAGCAACATTTCCCGTGGTGCTTTCATTGGACTGCGCAACTTGGTGACACTT
GACCTGTCAGACAACAGTATAACACATCTGGAAGATGGAACGTTGATTGGAATGCCCAAA
CTAGAAACACTGGATCTAAGTGGTAACACCATTAAAACCATTGGCAACAGTGTCTTCAAG
ACTGTAAGATCACTAAAGAGATTAACAACTGATGAATATAGATTCTGCTGCTTGGCACAG
CATGTTAGTGTGTGCAAGCCACCACCTGATGAGTTTTCCTCATGTGAAGATCTTATGTCT
AACTTGGTGCTTCGTGTCTGTGTGTGGATCTTGGGGATCATGGCAACTTTGGGAAATACA
CTGGTTATTATCTGGAGAATGTGTTTCAGGCATCCTAATAAGGTTCATTCTTTCCTTATC
ACCAATCTTGCCATAGGTGATTTATTAATGGGAGTTTACCTTCTCATAATTGGAAGTGCT
GATACTTACTACCGTGGAGTTTATTCAGTTCATGATCAGAACTGGAGAAGTAGTGGATTG
TGCCATACAGCAGGATTTTTGAGCACCTTTTCCAGTGAATTTTCAGTGTACACATTAACT
GTTATTGCCTTGGATCGATTTTTGGTGATACTATTTCCATTTCATGTGAGAACAATGAAA

ATGACAAAAACTAGATTATTGATGGCAGGAGGTTGGTTGGGTGCTGCCCTTTTGTCAGGT
TTACCTTTTATACACATCAGCTACTTTAAGAATTTTTATGGAAGATCTGGAGTTTGCCTT
GCACTTCATATAACTCCACAGAAATATCATGGATGGGAATATTCAGTGTTTGTATTCCTC
TTTTTGAATTTAGCATCTTTCTCAGTTATTGCTGTTGGCTATACTTCAATGTTTGTGATA
GCAAAGACAACTCAACAAGCAACACAAATAAATCAGAAAACTGCCGAGTCAGCTGTAATT
GCACGCCGCATGACTCTGATTGTTGCAACAGATGCAGCTTGCTGGATGCCTATCATATTC
TTGGGAATATTGTCTTTAGCAGGAACAACAGTCCCTCCACAGGTTTTTGCGTGGATAGCA
GTATTTGTTCTACCACTAAATGCAGCTATCAATCCTATTCTTTACACAATATCAACTACA
CCTTTCCTGGAGCCTACTTTGAAACAATTGAATACTTTGAAGAGATCATGCAGGCTGTCT
CTTACAACAGAACAGCACCACACATATTCCTTTGTTACAGCCTCAGAACACCAACATGAT
TTTAACCCCAGTGCCCAACAATTTATTATTGACACTGGGTATTCTGTTGGAAACGGCAAA
GTCAAAAAGAAACAACAACAAAATTGTTCTGATTCCAGTGAAGAGGATGGAGAACATACC
TGTAAACGCCAACAGCATCTGCACCTCACTTCTCAGAACTATCACACAGAAGCATCATGT
CATCACTCGCACCCACCGACTGAGTTGTTTATATCCACATCACTCTGCAATCTTAACACT
AATGCACATGCTCAGGTTGAACTTCTACCGCTGCAACAAATGGCTGTGGATAAATGA

>*Locusta migratoria* LGR5a

MPHYCRTVCLCLLMLLLSAPFSEAAEIERESTAAEPSRWQCSSTQEQPGRQEVSCGDGWC
VAEGALCNGVPDCAGGQDEAVHHCGCLPNEFRCSNSCIDLVRRCDREVDCPTGEDEDDCE
SFVCPVTHFKCANHFCVPEDAVCNFEDDCGDGSDELSCSHRSCWNSEFRCDNGECMRPGF
VCDGIVDCKDASDEAYCSPAEFRLCGDGTRVHKFFWCDGWPHCHDNHADELNCSECNGAG
EYLCPNGRCIRRANVCDSYCDCVPTARGQPCADEVDCGSKYVKQHGVSLCIPGETLSCFL
PSTKKYRDERCISSDFICDGYNDCHNGRFISDEFGCDMLTSSYPEDVFNCSEGRGIPVSL
VCDFKRDCLNGEDEQFCGKRDCSVKEYTCASGQCIPLEQVCDLNFDCWDKTDEMNCSNLR
CSEGYQRCRYSGQCIPAARWCDYSVDCFDGSDEEECESVQRDCRSDEFRCNSGQCIFADL
QCFKPGTEHTACADGSHLINCDNWTCPDGTFKCRNGPCLNMSLLCNKKIDCKDTWVDEDH
CPFECCGDNRCFCLDTKINCSSLELRDLPYGIEVQINRFYMANNLLNYSLNVDTFSKLDR
LLYLDLTNNSISWLPPLMFRNLWRLHILNLQNNKLSVISNGTFFGLPNLGTLHLHGNNIQ
SLEPMGFYGLSSLKALDLRHQRISNISRGAFIGLRNLVTLDLSDNSITHLEDGTLIGMPK
LETLDLSGNTIKTIGNSVFKTVRSLKRLTTDEYRFCCLAQHVSVCKPPPDEFSSCEDLMS
NLVLRVCVWILGIMATLGNTLVIIWRMCFRHPNKVHSFLITNLAIGDLLMGVYLLIIGSA
DTYYRGVYSVHDQNWRSSGLCHTAGFLSTFSSEFSVYTLTVIALDRFLVILFPFHVRTMK
MTKTRLLMAGGWLGAALLSGLPFIHISYFKNFYGRSGVCLALHITPQKYHGWEYSVFVFL
FLNLASFSVIAVGYTSMFVIAKTTQQATQINQKTAESAVIARRMTLIVATDAACWMPIIF
LGILSLAGTTVPPQVFAWIAVFVLPLNAAINPILYTISTTPFLEPTLKQLNTLKRSCRLS
LTTEQHHTYSFVTASEHQHDFNPSAQQFIIDTGYSVGNGKVKKKQQQNCSDSSEEDGEHT
CKRQQHLHLTSQNYHTEASCHHSHPPTELFISTSLCNLNTNAHAQVELLPLQQMAVDK*

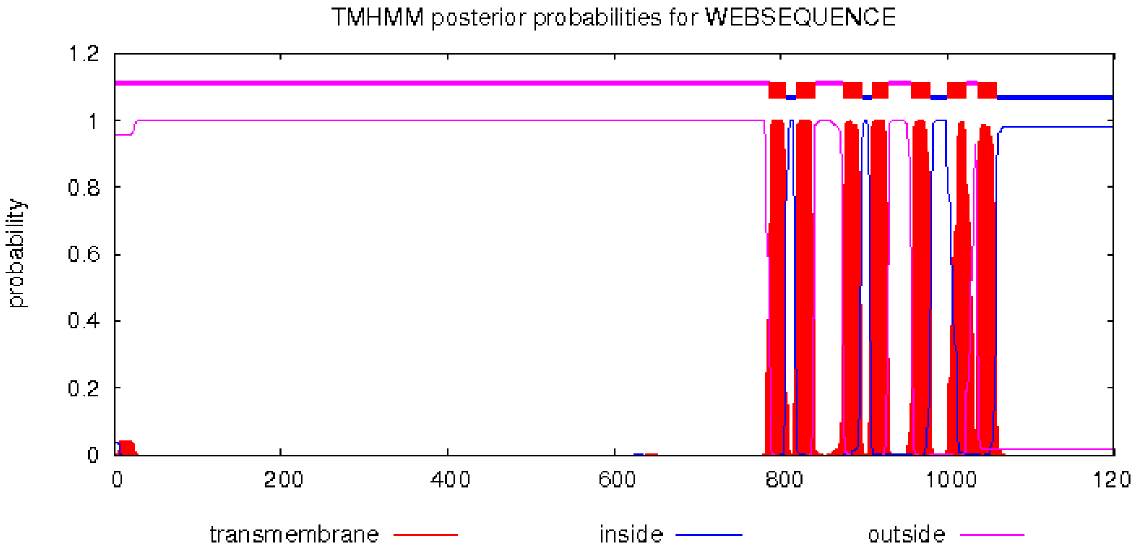

Transmembrane regions of *Locusta migratoria* LGR5a as determined at:
http://www.cbs.dtu.dk/services/TMHMM/

>*Locusta migratoria* LGR5b coding sequence Note: incomplet 5’-end

GCGTCGCTGGCGGCGGCGGCCGGCGTCGCGGCGCCGCCGGCTCAGTGCGTCCGCGGCTGT
GCGCCGCCCCCAGACGCTCCTGCTGACGCTGCCGAAGCCGCCGACCACGGACTCCAACGC
CAGCGCGCGGACTCGCTGTTGGTGGCGCACCAGCTACGAGGTGTGACTGGCGCTACGCAC
TCCACCAGGTCCTTGGCGGCAGTGGTTGGGGAAGAGTCCGCAGCGGCGCTGTCCGCCATG
TGGCTGGTGCTGGCGCTGCTGCTGACGACGTCGACGACGCGCGCGGGCGCCACCACCGCC
ACAGGCGGTGACCACGGCAACGACAGCACCGGCGTCGGAGGGCCCACGTGGTGGCCGTGC
AACGCGACGGCGCCCGGCGAGGAGGGAGGCGTGGGCGGCGTGGGCGGCGGGCAGGCGGCC
TTCAGCTGCGGCGGCGGCCGCTGCGTGGCGGCGCGCGCCCTCTGCAACGGCGTGCCGGAC
TGCCCCGCCGGCGAGGACGAGAGCGTGCACCACTGCGGTTGCCTCCCAAATGAGTTCCGG
TGCAGTAACTCCTGCATAGACCTGGTGCGGCGCTGTGATCGCGAACCCAACTGCCCTGAT
CAGGAGGACGAAACTGGCTGTGAATCCTTCGTGTGTCCTGTGACGCATTTCAAGTGTGCC
AACCACTACTGCGTGCCGGAGGATGCCGTGTGTAACTTCGAGGACGACTGTGGCGATGGG
TCGGATGAACAGAATTGTTCGCACCGGACGTGCTGGAACTCGGAGTTCCGGTGCGACAAC
GGGGAGTGCATCCGGCCCGGCTACGTCTGCGACGGCCTCGTCGACTGCAAGGACGCCAGC
GACGAGGCGCACTGCGCTCCAGATTTCTTCCGGGTGTGCGGCGACGGCAGCCGGGTGCAC
CAGTTCTTCTGGTGCGACGACTGGACGCACTGCGCCGATAACCACGCCGACGAGCTCAAC
TGTACGGCTTGCGACGGCGCCGACGAGTTCCGGTGCCCCAACGGGCGGTGCATCCGCAGG
GCCAACCTGTGCGACTCCTACTGCGACTGCGTGCCCACCGGCCGCGGGCAGGGCTGCGCC
GACGAGGCGGGCTGCCACCACACCTACCTGCTCAGCCACGGAGTGAGCTTGTGCACGCCT
GGCAAGACGCTGGTGTGCACGCCGCACCCCGGCAGCCGCGACGACCGCTGCATCGCGCCG
CGCTACATCTGCGACGGACACAACGACTGCCACAACGGCAACTACGTCTCCGACGAGCTG
GGCTGCCTGCCGTCGGCCGGGCTGGTGTCGGGCCAGTGGTTCTCGTGCTCGGAGGGCCGC
ATGGTGCCCCAGGAGACTCGCTGCGACTTCAAGTGGGACTGCCTGCTGGGCGAGGACGAG
CACGGCTGCGAACGGGTGCCGTGTTCTGAACGTGAATTCACATGCAGCAATGGCCAGTGT
ATTCCGATTGAAAGCCGTTGTGATCTAAAATTTGATTGCTGGGACAAGAGCGATGAATTT
AACTGCAAGGAGGCGCCGTGCCCGGCGGGCCACCTGCGGTGTCGCGACAGCGCGCAGTGC
GTGCCGAGAGCCTGGTGGTGCGACTTCTACCTCGACTGTCCCGACGGCTCCGACGAGACC
AGCTGCGAGGGCCGCGGGCGGGAGTGCGGCGCTGAGGAGTTCCGCTGCCAGAGCGGGCAG
TGCGTGCCGCGCCGCCACGTCTGCTTCAAGTCCGGCCAGGACCGCAGCGGCTGCGCCGAC
GGCTCACACCTGCTGCACTGCGAAAATGTGACGTGCCCCGACAACACATTTAAATGCAGA
AACGGGCCGTGCCTCAACATGTCGCTGCGCTGTAACGCCGTCCTTGACTGTAAAGACACC
TGGGTTGATGAAGACGGCTGCACGTTCCAGTGCTCCACGGAACCTCGCTGTGTGTGCCAC
GACACCAAAATCAACTGCACGAGCCTTGAGCTGAAACAGTTCCCGGCAGAAATTGAATTC
CAGATAAATCGCTTCTACATGGCAAATAATTTGTTGAATGAAACACTCAACGAAGATTCC
TTTTCGAAATTGGATCGACTTGTTTACTTAGATTTGAGGAACAACAGCATATCTAGGCTT
CCCGCCATGATATTCCGTAATCTCTGGAGACTACATATTCTCAATCTGCAAGATAACCGT
ATATCCGTTATAAATAATGGTAGCTTCTTCGGATTACCGGATTTGCGCACTCTCCATCTA
CAAGGCAACGGCATAGAAGTCTTAGAGCCAATGGCGTTTTACGGACTGTCATCGATTACC
ACCTTGGATCTGAAGCACCAGAGGATACGTAACGTATCTCGCAACGCTTTCGTTGGCCTC
AGAAGTCTTGTTGCGCTTGACCTGTCTTTCAACAACATATTCTACCTTGCAGACGGATCG
TTGAATGGAATGCCTAAACTGGAAACACTAGATCTCAGTAATAATTTCATCAAGTACGTT
GGAAACAACGTGTTTAGAAGTGTAACTGCATTGAAAACACTGGTGACGGACGAGTTCCGC
TTCTGCTGCGTGGCGCGGCACGCCGCGCGCTGCCTGCCCGCGCCCGACGAGTTCTCGTCG
TGCGAGGACCTCATGTCCAACCTGGTGCTGCGCGTCTGCGTCTGGGTGCTCGGCGTCGTC
GCCACCGCCGGCAACGCGCTCGTCATCGCCTGGCGCATGCGCTTCAAGCACTCCAACCAG

GTGCACTCGTTCCTGATCACCAACCTGGCCATCGGCGACCTGTTCATGGGCTCGTACCTG
CTGATCATCGCGGCGGCGGACGCGCACTACCGCGGCGTCTACTCGGTGCACGACCGCGAC
TGGCGCTCCAGCGCGCTCTGCAACCTGGCCGGCTTCATCTCCACCTTCTCCAGCGAGTTC
TCCGTCTACACGCTCACAGTGATCACGCTGGACCGCTTCCTGGTGATAATCTTCCCGTTC
CGCATCCGGAGGCTGGAGATGTCCAAGACGCGCGTGCTCATGGCCGCCGGCTGGGCCGTG
GCCGCCTTCCTGTCCGGGCTGCCGCTCTTCCGGATAGACTACTTCCGGAATTTCTACGGG
AGATCCGGCGTTTGCTTAGCCCTGCACATAACTCCCGACAAGCCGAACGGATGGGAGTAC
TCCGTATTTGTATTCCTGTTCCTGAACCTGGCGTCGTTCTCGGCGATCGCGCTGGGCTAC
GCCTGGATGTACTGGGTGGCGCGGTCGACGCAGCGCGCGGCGCGCCACCAGCACCGCCGG
CCCGACTCGGCCACCATGGCGCGCCGCATGACGCTCATCGTGGCCACCGACGCCGCCTGC
TGGATGCCCATCATCCTGCTCGGCCTGCTCTCGCTCGCCGGCATCACCGTGCCGCCGCAG
GTGTTCGCGTGGGTGGCGGTGTTCGTGCTGCCGCTGAACGCGGCGGTGAACCCGGTGCTG
TACACCATCTCGACGGCGCCGTTCCTGACGCCGGCGCGCCGCGGCCTGCGCGCCTTCAAG
CGCTCCTGGAAGCTGTCGCTCACCGCCGACCAGCGCAGGACCTTCTCCTCCTCGCTGGGT
TCGTCCCACCTGCACAACTGCAACCTGTGCGACGTGAACTGCCCGCTAGACGTCAACATC
TTCATGGAGAACGGCGGCGGCGTTGCCACCCCCGCGGAACGAACCGAGTTCGCTGGCGGG
AGCTCCAATAACGGGTCGCAGCACTTCGCGCTCTTCTCGCTCACCAGACGGCAGCCGCAG
GCGTCACACGATCACCGGTGGTGCTCCCGGCCTGCGGCGCAGCAGCGGCACGGCAGCAGC
GGCGACTGGCGCTTCGGTCGGTGGCGCGAGCAGCAGCCTCAGCAGTCGCTGGCGGCCGTC
GACACGGCTGTCTCTGCGCGCGGCGAGGTCATCCCCCTGCGCCAGGTGGCCGCCGCCGCC
CCCGCTGCCGCCCCTGCGAATGACTCCCGCCGCCGCCGCCTCCGCGAGCTGTACGACTGA

>*Locusta migratoria* LGR5b Note: incomplete N-terminus

ASLAAAAGVAAPPAQCVRGCAPPPDAPADAAEAADHGLQRQRADSLLVAHQLRGVTGATH
STRSLAAVVGEESAAALSAMWLVLALLLTTSTTRAGATTATGGDHGNDSTGVGGPTWWPC
NATAPGEEGGVGGVGGGQAAFSCGGGRCVAARALCNGVPDCPAGEDESVHHCGCLPNEFR
CSNSCIDLVRRCDREPNCPDQEDETGCESFVCPVTHFKCANHYCVPEDAVCNFEDDCGDG
SDEQNCSHRTCWNSEFRCDNGECIRPGYVCDGLVDCKDASDEAHCAPDFFRVCGDGSRVH
QFFWCDDWTHCADNHADELNCTACDGADEFRCPNGRCIRRANLCDSYCDCVPTGRGQGCA
DEAGCHHTYLLSHGVSLCTPGKTLVCTPHPGSRDDRCIAPRYICDGHNDCHNGNYVSDEL
GCLPSAGLVSGQWFSCSEGRMVPQETRCDFKWDCLLGEDEHGCERVPCSEREFTCSNGQC
IPIESRCDLKFDCWDKSDEFNCKEAPCPAGHLRCRDSAQCVPRAWWCDFYLDCPDGSDET
SCEGRGRECGAEEFRCQSGQCVPRRHVCFKSGQDRSGCADGSHLLHCENVTCPDNTFKCR
NGPCLNMSLRCNAVLDCKDTWVDEDGCTFQCSTEPRCVCHDTKINCTSLELKQFPAEIEF
QINRFYMANNLLNETLNEDSFSKLDRLVYLDLRNNSISRLPAMIFRNLWRLHILNLQDNR
ISVINNGSFFGLPDLRTLHLQGNGIEVLEPMAFYGLSSITTLDLKHQRIRNVSRNAFVGL
RSLVALDLSFNNIFYLADGSLNGMPKLETLDLSNNFIKYVGNNVFRSVTALKTLVTDEFR
FCCVARHAARCLPAPDEFSSCEDLMSNLVLRVCVWVLGVVATAGNALVIAWRMRFKHSNQ
VHSFLITNLAIGDLFMGSYLLIIAAADAHYRGVYSVHDRDWRSSALCNLAGFISTFSSEF
SVYTLTVITLDRFLVIIFPFRIRRLEMSKTRVLMAAGWAVAAFLSGLPLFRIDYFRNFYG
RSGVCLALHITPDKPNGWEYSVFVFLFLNLASFSAIALGYAWMYWVARSTQRAARHQHRR
PDSATMARRMTLIVATDAACWMPIILLGLLSLAGITVPPQVFAWVAVFVLPLNAAVNPVL
YTISTAPFLTPARRGLRAFKRSWKLSLTADQRRTFSSSLGSSHLHNCNLCDVNCPLDVNI
FMENGGGVATPAERTEFAGGSSNNGSQHFALFSLTRRQPQASHDHRWCSRPAAQQRHGSS
GDWRFGRWREQQPQQSLAAVDTAVSARGEVIPLRQVAAAAPAAAPANDSRRRRLRELYD*

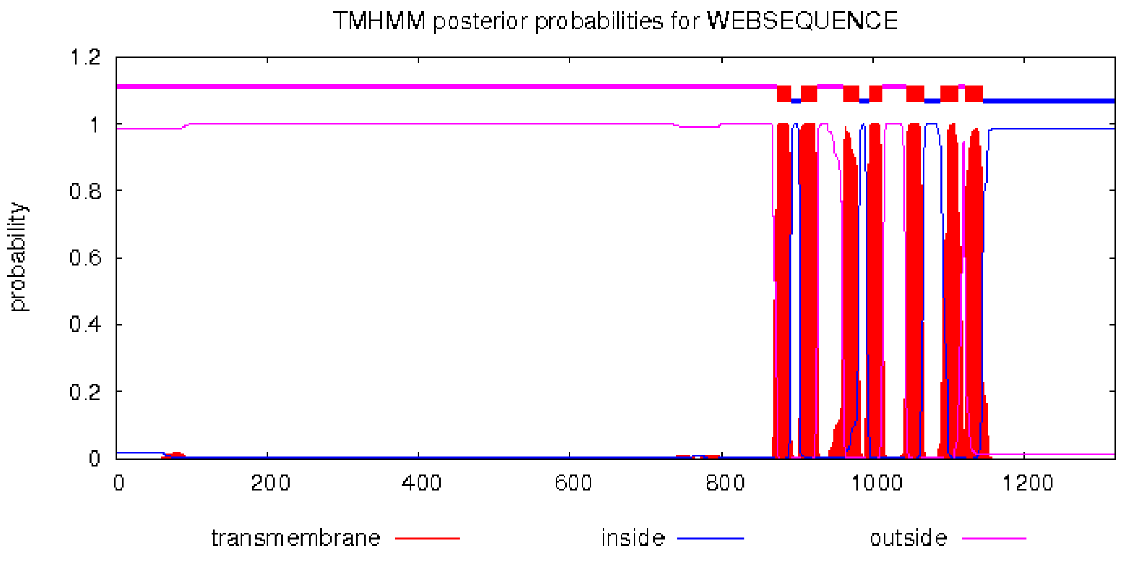

Transmembrane regions of *Locusta migratoria* LGR5b as determined at:
http://www.cbs.dtu.dk/services/TMHMM/

>*Schistocerca gregaria* LGR5a coding sequence

ATGACTGACCTCTGCAGCGCCGTTCGTCTTCTCCTGCTACTGCTCTTGTTGGGTGCATCC
TGCTCCGAGGCCGCTGACACAGGAAGCCAGTCTACTACGGCCGTGGAAGCGTCGCGCTGG
CTGTGCAACAACACGCAGGTGGAGCCTGGAGGGCGGGAGGTGAGCTGCGGCGACGGCTGG
TGCGTGGCGGAGAGTGCTCTGTGCAACGGCGTCCCGGACTGCGCCGGCGGCGAGGACGAG
GGCGTGCCGCACTGCGGATGCCTGCCCAATGAGTTCCGGTGCAGTAATTCGTGCATCGAC
CTCGTGCAGCGCTGCGACCGCGACGTCGATTGCCCGAACGGCGAAGATGAGGACGGCTGC
GAGTCATTCGTCTGCCCAGTGACGCACTTCAAATGCGCCAACCACTTCTGTGTGCCTGAG
GACGCCGTCTGCAACTTCGGAGACGACTGCGGTGACGGCTCGGACGAACAAAGCTGCTCG
CACCGGTCTTGCTGGAAGTCGGAGTTCCGGTGTGACAACGGAGAGTGCATCCGTCCTGGC
TTCGTGTGCGACGGCGTCGTCGACTGCAAGGACGCCAGCGACGAGGTGCACTGTACACCA
GCCGAGTTCCGCATTTGTGGCGACGGAACGCGAGTTCACAAGTTCTTCTGGTGTGATGGT
TGGCCGCACTGCCACGACAACCACGCCGACGAACTCAACTGCACCGAGTGCAGAGGTCAG
GACGAATATCTCTGTCCAAACGGCCGCTGCATCAGGCGCGCCAATGTCTGCGACTCATAC
TGCGATTGCGTTCCGGCTTCCAGAGGGCAACCATGCGCCGACGAGGTGGACTGCGCAGGC
AGATATCTCAAGGAGTACGGAGTCAGTGTCTGCACACCAGGTGAAACATTGTCGTGTGTT
CTGCCATCCAACAAGAAGCACCGAGAGGAACGTTGTATCTCATCAGACTTTATATGTGAC
GGCTATAATGACTGTCATAACGGAAGATATATCTCCGATGAATTTGGGTGTGAAGATCTG
TTCAATTGTTCTGAGGGACGTGCTATACCCCTGTCCCTCGTGTGTGATTTCAAACGAGAC
TGTCTGAATGGAGAAGATGAACAGTTCTGTGGAAAAAAGAGTTGCTCAGTAAAAGAATTC
ACTTGTGCTAATGGACAATGTATTCCATTGGAGCTGGTCTGTGATCTGAGTTTTGATTGT
TGGGATAAGACTGATGAAATGAACTGCACAAATCAGAAGTGTTCTCAAGGATATCGGAAA
TGTAGATACAATGGTCAGTGCATCCCTTTAACTTGGTGGTGTGATTACAGTATAGACTGC
TTTGATGGCAGTGATGAAGAGGAATGTGAATCTGCACAGCATGCTTGCAAATCCAATGAA
TTTCGCTGCAACAATGGTCAATGTGTACCTGCTGACCTACGGTGTTTCAAATCCGGTATT
GATCATGCTGCATGTGCTGATGGATCTCATCTGTTTAACTGTGATAACTGGACGTGCCCT
GATGGGACTTTCAAATGTAGAAATGGTCCTTGCCTGAACAATTCTTTAGTGTGCAATAGG
AACATTGATTGCAAAGATACTTGGGTGGATGAAGATCACTGCCCATTTGAATGCTGTAGT
GACAGTCGCTGTTTCTGTCTGGACACAAAAATTAACTGCAGTACTCTTGGCCTGAAGACT
CTCCCAGATGGAATTGAAATTCAAATAAATCGCTTCTACATGGCCAATAACTTGCTGAAC
TACTCACTCAATGTTGATACCTTCTCAAAACTAGACCGGCTGTTATACTTGGATCTGAGA
AACAACAGTATTTCATGGCTACCTCCAATGATGTTTCGTAATCTGTGGCGACTGCACATC
CTCAATTTACAGAATAACAACTTATCTGTAATTAGCAATGGTACATTCTTTGGATTACCT
AAACTTGGAACACTTCATCTACATGGTAACAACATCCAGAGCTTAGAGCCAATGGCCTTT
TATGGTCTCTCTTCACTGAAAGCACTTGATCTTAAACGCCAAAGGATAAGCAACATTTCC
CATGGTGCTTTCATTGGGCTACGTAGCTTAGTGACACTTGACATGTCATACAACTGTATA
GCTTATCTAACAGATGGAACACTGATTGGAATGCCCAAGCTGGAGACACTGGATCTGAGT
GGTAACAGCATTAGAACTGTTGGCAGTACTGTCTTCAAGAGTGTAAGGTCCCTGAAGAGA
TTAACAACTGATGAATACAGATTCTGCTGCTTGGCACGACATGTTAGTGTCTGCATGCCA
CCACCTGATGAGTTTTCTTCATGTGAAGATCTTATGTCTAATTTGGTACTTCGTGTATGT
GTATGGATCCTTGGCATCGTGGCAACTTTAGGAAACACATTGGTCATTGTGTGGAGAAAG
TGTTTCAGGCATCCTAATAAGGTTCATTCGTTCCTTATCACCAATCTTGCTATAGGAGAT
TTATTAATGGGAGTTTACCTTCTCATAATTGGAAGTGCTGATACTTACTACCGTGGAGTT
TATTCCGTTCATGATCGTGACTGGAGAAGTAGTGGATTGTGTCATACTGCAGGATTTCTT
AGCACCTTCTCTAGTGAATTCTCAGTGTATACACTCACTGTTATTGCTTTGGATAGATTC
TTGGTGATACTATTTCCATTTCATGTGAGGACAATGAAAATGACAAAAACCCGAATATTG

ATAGCAGGAGGATGGTTGGGTGCTGCATTTTTATCAGGTCTACCGTTTATACACATCAGC
TATTTTAAGAATTTTTATGGAAGATCTGGAGTTTGCCTTGCACTTCACATAACACCACAG
AAATATAATGGATGGGAATATTCAGTGTTTGTATTCCTCTTCTTGAATCTGGCATCATTC
TCTGTTATTGCTGTTGGGTATACGTCAATGTTTGTGATAGCGAGGACAACTCAACAAGCA
GCACAAATGAATCAGAAAACTGCTGAGTCAGCAGTGATTGCCCGCCGAATGACTCTTATT
GTTGCTACAGATGCAGCTTGCTGGATGCCTATCATATGCTTGGGGATACTGTCTTTGGCA
GGAACGACAGTCCCTCCACAGGTTTACGCATGGATAGCTGTATTTGTTCTACCACTAAAC
GCAGCTATCAATCCTATTCTTTACACAATATCAACTACACCTTTCCTAGAACCAACTCTA
AAAGGACTGAAGACTTTGAAGAGGTCATGTAGGCTGTCGCTTACAACTGAACAGCACCAC
ACATACTCATCTGTTACAGCCTCTGAACATCAACATGATTATAATTTCCATGGCCATCCA
TTTATGATTGATGCTGGTCATTCAATTGGAAACGACAAATACAAAAAGAAACAACAAGAA
AATTGTAATGATACCAGTGAAGAGGACGGAGAACATTTCTTTTCTGCCAGTGAATGCCAA
CAGCATCTACACCTCACTTCTAAGAACTGTCACACAGAAGCAATGTGTCATTCCTCCCCA
TCAGCTGAGTTGGTGATGTCCAACTCACACTTCAATGAGAGATGTACTTACCCTGACATC
CAAGTTCAGGTTGAACTTCTACCATTGCAACAAATGCCTGTGGATATGTGA

>*Schistocerca gregaria* LGR5a

MTDLCSAVRLLLLLLLLGASCSEAADTGSQSTTAVEASRWLCNNTQVEPGGREVSCGDGW
CVAESALCNGVPDCAGGEDEGVPHCGCLPNEFRCSNSCIDLVQRCDRDVDCPNGEDEDGC
ESFVCPVTHFKCANHFCVPEDAVCNFGDDCGDGSDEQSCSHRSCWKSEFRCDNGECIRPG
FVCDGVVDCKDASDEVHCTPAEFRICGDGTRVHKFFWCDGWPHCHDNHADELNCTECRGQ
DEYLCPNGRCIRRANVCDSYCDCVPASRGQPCADEVDCAGRYLKEYGVSVCTPGETLSCV
LPSNKKHREERCISSDFICDGYNDCHNGRYISDEFGCEDLFNCSEGRAIPLSLVCDFKRD
CLNGEDEQFCGKKSCSVKEFTCANGQCIPLELVCDLSFDCWDKTDEMNCTNQKCSQGYRK
CRYNGQCIPLTWWCDYSIDCFDGSDEEECESAQHACKSNEFRCNNGQCVPADLRCFKSGI
DHAACADGSHLFNCDNWTCPDGTFKCRNGPCLNNSLVCNRNIDCKDTWVDEDHCPFECCS
DSRCFCLDTKINCSTLGLKTLPDGIEIQINRFYMANNLLNYSLNVDTFSKLDRLLYLDLR
NNSISWLPPMMFRNLWRLHILNLQNNNLSVISNGTFFGLPKLGTLHLHGNNIQSLEPMAF
YGLSSLKALDLKRQRISNISHGAFIGLRSLVTLDMSYNCIAYLTDGTLIGMPKLETLDLS
GNSIRTVGSTVFKSVRSLKRLTTDEYRFCCLARHVSVCMPPPDEFSSCEDLMSNLVLRVC
VWILGIVATLGNTLVIVWRKCFRHPNKVHSFLITNLAIGDLLMGVYLLIIGSADTYYRGV
YSVHDRDWRSSGLCHTAGFLSTFSSEFSVYTLTVIALDRFLVILFPFHVRTMKMTKTRIL
IAGGWLGAAFLSGLPFIHISYFKNFYGRSGVCLALHITPQKYNGWEYSVFVFLFLNLASF
SVIAVGYTSMFVIARTTQQAAQMNQKTAESAVIARRMTLIVATDAACWMPIICLGILSLA
GTTVPPQVYAWIAVFVLPLNAAINPILYTISTTPFLEPTLKGLKTLKRSCRLSLTTEQHH
TYSSVTASEHQHDYNFHGHPFMIDAGHSIGNDKYKKKQQENCNDTSEEDGEHFFSASECQ
QHLHLTSKNCHTEAMCHSSPSAELVMSNSHFNERCTYPDIQVQVELLPLQQMPVDM*

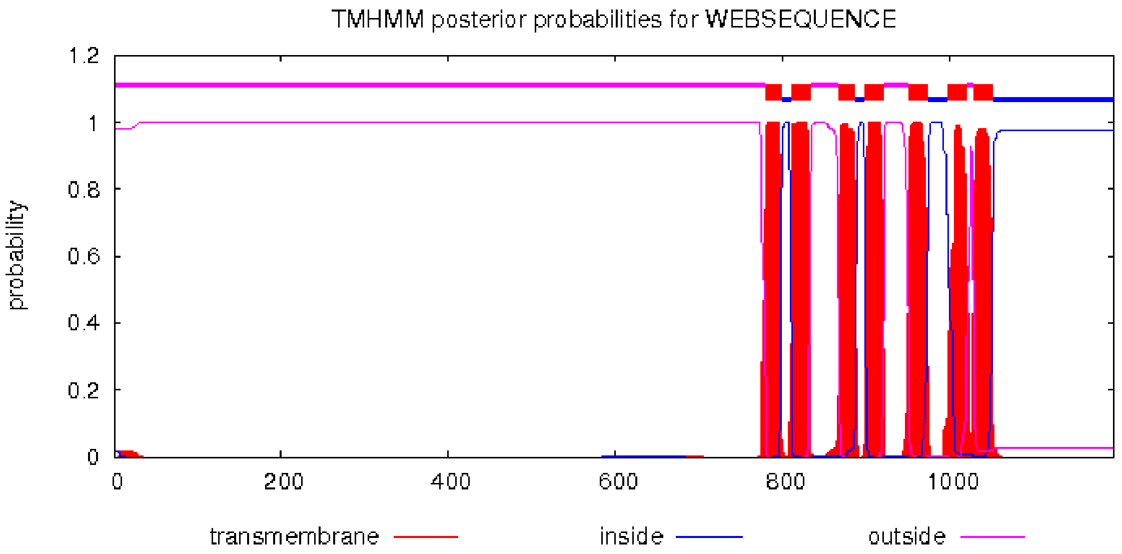

Transmembrane regions of *Schistocerca gregaria* LGR5a as determined at:
http://www.cbs.dtu.dk/services/TMHMM/

>*Schistocerca gregaria* LGR5b coding sequence

ATGTGGCGCCTGCTGCTGCTGACTTTGGCCACGAGAGCGGCTGCGCTCACCGCCAGTGGG
GGTGACCACGGCAACGACAGCGCTGGCGGCGGAGGGCCGACGTGGTGGTCGTGCAACGCG
ACGTCGCCCGGCGAGGAGGGGCGCGCCACGGAGGCGGCGGCCGTGTTCAGCTGCGGCGGC
GGCTGGTGCGTGGCGGAGCGAGCTCTCTGCAACGGCGTCCCCGACTGCCCCGCGGGCGAG
GACGAGAGCGTCCACCAGTGCGGTTGCCTCCCAAATGAATTCCGGTGCAGTAACTCTTGC
ATCGACCTGGTACGACGCTGTGATCGCGAACCCAACTGCCCTGATCAGGAGGACGAAACC
GGCTGTGAGTCCTTCGTGTGTCCTGTGACGCACTTCAAGTGTGCCAATCACTACTGCGTC
CCAGAGGACGCGGTGTGCAACTTTGAGGACGACTGTGGTGATGGATCCGACGAACAAAAT
TGCTCGCACCGGACATGCTGGAACTCGGAGTTCCGGTGCGACAACGGGGAGTGCATCCGG
CCCGGCTACGTCTGCGACGGCGTCGTCGACTGCAAGGACGCCAGCGACGAGGCGCACTGC
ACGCCAGATTTCTTCCGGATATGCGGCGACGGGACTCGCGTCCACCGCTTCTTCTGGTGT
GACGACTGGACGCACTGCGCTGATAACCACGCGGACGAGCTGAACTGTACAGCCTGCGAC
GGCGCCGACATGTTCCGCTGTCCGAACGGTCGCTGCATCCGTCGCGCCAACCTGTGTGAC
TCGTACTGCGACTGCGCGCCCACCGAACGCGGCCAGGGGTGCGCCGACGAGGCGGGCTGC
CAGCACAGCTACCTCCAGAGGCACGGTATGAGCGTGTGCACGCCGGGGAAGAGCTTACTT
TGCATCCCGCACCCCGGCAGCAGGGAGGATCGCTGCATCGCCCCCGAGTACATCTGCGAC
GGCCACAACGACTGCCACAACGGGAACTACGTCTCGGACGAGCTGGGCTGCCTGGCTGAC
GTGCTCCGAGGGCCGCATGATCCCGCAGGAGCTGCGCTGCGACTTCAAGTGGGATTGCCT
GCTGGGCGAGGACGAGCAGGGTTGCAACGGATCCCGTGTTCTAAAAACGAATTCACGTGC
AGCAATGGCCAATGTATCCCTATAGAAAGCCAATGTGATTTGAGATTTGACTGCTGGGAC
AAGAGTGACGAATTTAACTGCAAGGAGGCGCCGTGCCCGCCGGGCCAGCTCAAGTGCGCG
GGCAGCGGGCAGTGCGTGCCGCGGGCCTGGTGGTGCGACTTCTACCTCGACTGCCCGGAC
GGCTCGGACGAGCTCAGCTGCGTGGGCCGCGGGCGGGAATGCGGCCGCGACGAGTTCCGC
TGCCTGAGCGGCCAGTGCGTGCCCCGCACCGACGTCTGCTTCAAGTCCGGCCACGAGCGC
AGCGGCTGCGCCGACGGCTCCCACCTGCTGCACTGCGAGAATGTGACGTGCCCAGAGGAC
ACATTTAAATGCAGAAACGGCCCGTGCCTCAACATGTCACTGCGCTGCAACGCCTTCCTG
GACTGCAAGGACACTTGGGTTGACGAAGACGGCTGCACGTTCCAGTGCTCCACGGAACCT
CGATGCGTGTGCCACGACACAAAAATCAACTGCACCAGCCTTGGTCTGACGGAGCTCCCG
GCGGTCATTGAATTTCAGATAAATCGCTTTTATATGGCAAATAATTTGTTGAATGAGACA
CTTAATGAAGATTCATTTTCGAAACTGGACCGACTTGTCTATCTAGATCTGAGGAACAAT
AGTATATCTAGACTTCCCGCCACGATATTCCGCAACCTCTGGCGACTACACATTCTCAAC
TTACAAGATAACCATATCTCCGTTATAAACAACGGTAGCTTCTTCGGGTTGCCAGATTTG
CGCACACTGCATCTACAAGGTAACGGAATAGAAGTCTTAGAACCAATGGCTTTTTACGGA
CTCTCGTCGATTACTACGTTGGATCTGAAACACCAGAGGATACGCAACATATCTCGCAAC
GCCTTCGTAGGACTCAGAAGTCTCGTCGCCCTTGACCTGTCTTTCAACCACATATTCTAC
CTTGCAGATGGAGCTTTGAATGGAATGCCTAAACTGGAAACATTAGACCTGAGTAACAAT
TTCATCAAGAACGTTGGAAACAACGTATTTAGAAGCGTAACTGCATTAAAAACACTGGTG
ACGGACGAGTTCCGGTTCTGCTGCGTGGCGCGGCACGCGGCGCGCTGCCTGCCGGCGCCC
GACGAGTTCTCGTCGTGCGAGGACCTCATGTCCAACCTGGTGCTGCGCGTCTGCGTGTGG
GTGCTCGGCGTCGTCGCCACCGCCGGCAACGCGCTCGTCATCGCCTGGCGCATGCGCTTC
AAGCACTCCAACCAGGTGCACTCGTTCCTGATCACGAACCTGGCGATCGGCGACCTGTTC
ATGGGGTCGTACCTGCTGATCATCGCGGCGGCCGACGCACACTACCGGGGCGTGTACTCG
GTGCACGACCGCGACTGGCGCTCCAGCGCCCTCTGCAGCCTCGCCGGCTTCATCTCCACC
TTCTCCAGCGAGTTTTCCGTCTACACGCTCACAGTGATCACGCTGGACCGTTTTCTGGTG

ATAATATTTCCGTTCCGGATCCGGAGGCTGGAAATGTCCAAGACCCGCATGCTGATGGCG
GCGGGCTGGGCGGTTGCAGCGTTCCTGTCCGGCTTGCCGCTCTTCAGGATAGACTACTTC
AGAAATTTCTACGGGAGATCCGGTGTTTGCCTAGCCCTGCACATAACTCCAGACAAGCCG
AGCGGATGGGAGTACTCCGTGTTTGTATTCCTATTCCTGAACCTGGCGTCGTTCTCGGTG
ATCGCGCTGGGCTACGTGTGGATGTACGCGGTGGCGCGCACCACGCAGCGCGCCGTCCAG
CAGCAGCACAAGCGGCCGGACTCGGCCACCATGGCGCGCCGCATGACGCTCATCGTGGCC
ACCGACGCCGCCTGCTGGATGCCCATCATCCTGCTCGGCCTGCTCTCGCTCGCGGGCATC
ACCGTCCCACCGCAGGTGTTCGCTTGGGTGGCGGTTTTCGTGCTGCCGCTGAACGCGGCG
GTCAACCCGGTACTGTACACCATCTCGACGGCGCCCTTCCTGACGCCGGCCCGGCGCGGC
CTGCGAGCCTTCAAGCGCTCCTGGAAGCTGTCGCTCACCGCAGACCAGAGGAGGACCTTC
TCTTCTTCGCTGGGTTCGACCCACGTCCACAACTGCAACCTGTGCGACGTAAACTGCCCG
CTTGACGTCAACATCTTCATGGAGAACGGCGGGGGCGTTGCCACCGCTCCGGAGCGTAGC
GATCTCGCTGGCGGGGGCGACAGTAACGACTCGCAGCACTTCGCCCTCTTCTCGCTCACC
AGACGCCAGCCGCAGGCGGCACACGATCACCGCTGGTGCTCTCGGTCATCCCAGCAGCGC
GCTAGCGGCGACTTGCGTTCCGGCCGATGGCGGGGGCGCCACCGTCAGCAGCTACACCTG
CAGCCGTCGCTGGCGTCGGTCGACACAGCTGTCTCTGCTCGCGGCGAGGTCATCCCCCTG
CGGCAGGTGGCCGGGCCCGCCCCCGCGGCTGACTCCCGCCGCCGCCGCCTCCGCGAGCTG
TACGACTGA

>*Schistocerca gregaria* LGR5b

MWRLLLLTLATRAAALTASGGDHGNDSAGGGGPTWWSCNATSPGEEGRATEAAAVFSCGG
GWCVAERALCNGVPDCPAGEDESVHQCGCLPNEFRCSNSCIDLVRRCDREPNCPDQEDET
GCESFVCPVTHFKCANHYCVPEDAVCNFEDDCGDGSDEQNCSHRTCWNSEFRCDNGECIR
PGYVCDGVVDCKDASDEAHCTPDFFRICGDGTRVHRFFWCDDWTHCADNHADELNCTACD
GADMFRCPNGRCIRRANLCDSYCDCAPTERGQGCADEAGCQHSYLQRHGMSVCTPGKSLL
CIPHPGSREDRCIAPEYICDGHNDCHNGNYVSDELGCLADVLRGPHDPAGAALRLQVGLP
AGRGRAGLQRIPCSKNEFTCSNGQCIPIESQCDLRFDCWDKSDEFNCKEAPCPPGQLKCA
GSGQCVPRAWWCDFYLDCPDGSDELSCVGRGRECGRDEFRCLSGQCVPRTDVCFKSGHER
SGCADGSHLLHCENVTCPEDTFKCRNGPCLNMSLRCNAFLDCKDTWVDEDGCTFQCSTEP
RCVCHDTKINCTSLGLTELPAVIEFQINRFYMANNLLNETLNEDSFSKLDRLVYLDLRNN
SISRLPATIFRNLWRLHILNLQDNHISVINNGSFFGLPDLRTLHLQGNGIEVLEPMAFYG
LSSITTLDLKHQRIRNISRNAFVGLRSLVALDLSFNHIFYLADGALNGMPKLETLDLSNN
FIKNVGNNVFRSVTALKTLVTDEFRFCCVARHAARCLPAPDEFSSCEDLMSNLVLRVCVW
VLGVVATAGNALVIAWRMRFKHSNQVHSFLITNLAIGDLFMGSYLLIIAAADAHYRGVYS
VHDRDWRSSALCSLAGFISTFSSEFSVYTLTVITLDRFLVIIFPFRIRRLEMSKTRMLMA
AGWAVAAFLSGLPLFRIDYFRNFYGRSGVCLALHITPDKPSGWEYSVFVFLFLNLASFSV
IALGYVWMYAVARTTQRAVQQQHKRPDSATMARRMTLIVATDAACWMPIILLGLLSLAGI
TVPPQVFAWVAVFVLPLNAAVNPVLYTISTAPFLTPARRGLRAFKRSWKLSLTADQRRTF
SSSLGSTHVHNCNLCDVNCPLDVNIFMENGGGVATAPERSDLAGGGDSNDSQHFALFSLT
RRQPQAAHDHRWCSRSSQQRASGDLRSGRWRGRHRQQLHLQPSLASVDTAVSARGEVIPL
RQVAGPAPAADSRRRRLRELYD*

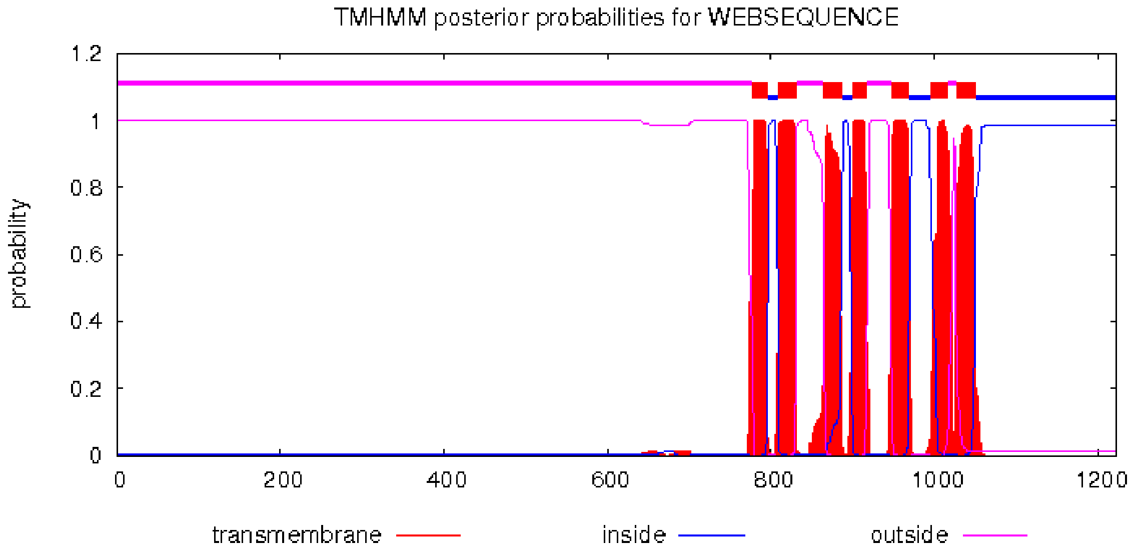

Transmembrane regions of *Schistocerca gregaria* LGR5b as determined at:
http://www.cbs.dtu.dk/services/TMHMM/
