# Supplementary figures and images for: Identification of Gonadulin and Insulin-Like Growth Factor From Migratory Locusts and Their Importance in Reproduction in Locusta migratoria
Source: Front Endocrinol (Lausanne). 2021 Jun 4;12:693068. doi: 10.3389/fendo.2021.693068 (PMC8220825; doi:10.3389/fendo.2021.693068)

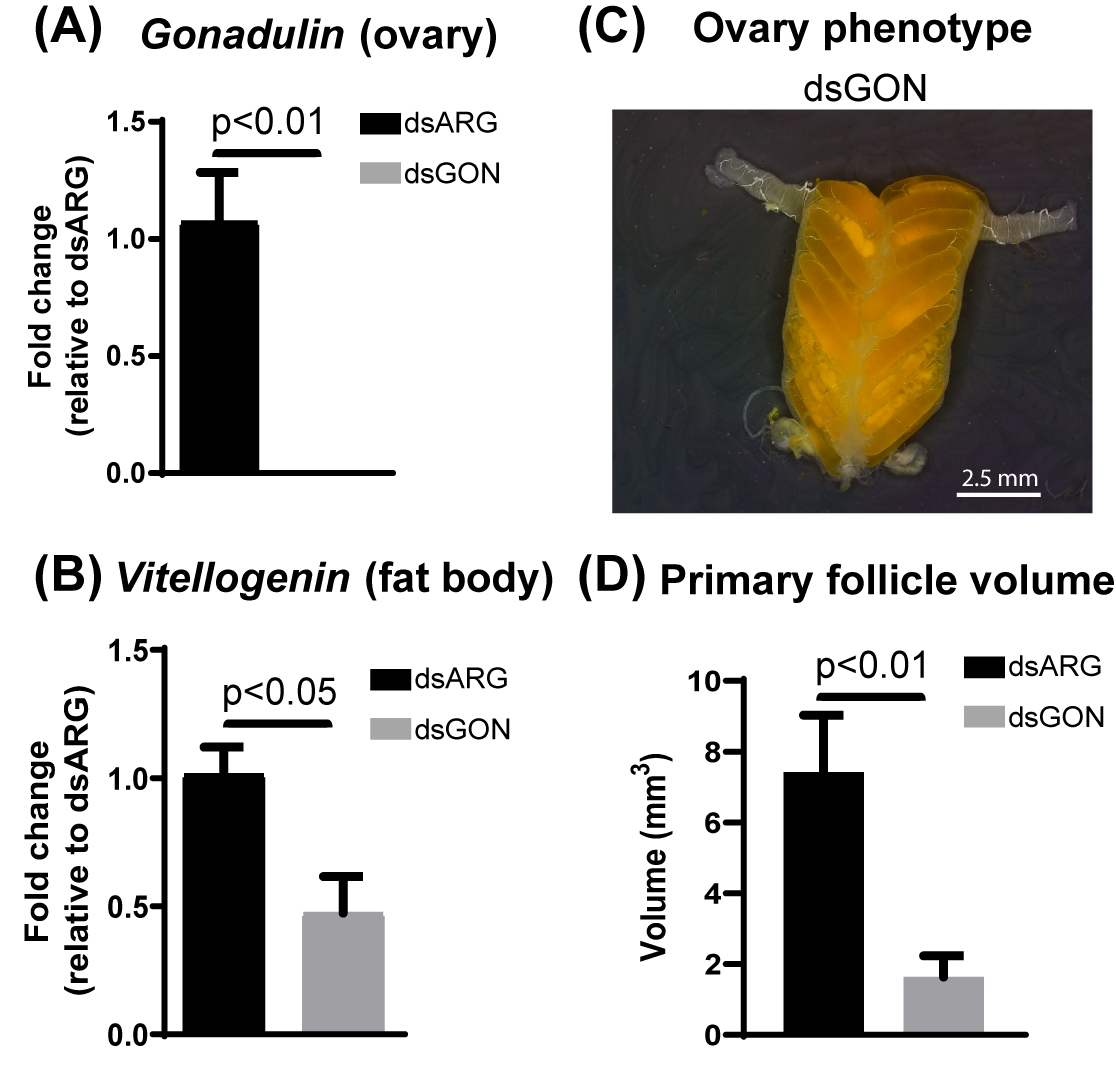

Supplement: Supplementary file 2 [file Image_1.tif]
